# Structural Heterogeneity in a Phototransformable Fluorescent Protein Impacts its Photochemical Properties

**DOI:** 10.1002/advs.202306272

**Published:** 2023-12-25

**Authors:** Arijit Maity, Jip Wulffelé, Isabel Ayala, Adrien Favier, Virgile Adam, Dominique Bourgeois, Bernhard Brutscher

**Affiliations:** ^1^ CEA CNRS Institut de Biologie Structurale (IBS) Université Grenoble Alpes 71 avenue des Martyrs, Cedex 9 Grenoble 38044 France

**Keywords:** fluorescence, PCFP, protein, solution NMR, super‐resolution microscopy

## Abstract

Photoconvertible fluorescent proteins (PCFP) are important cellular markers in advanced imaging modalities such as photoactivatable localization microscopy (PALM). However, their complex photophysical and photochemical behavior hampers applications such as quantitative and single‐particle‐tracking PALM. This work employs multidimensional NMR combined with ensemble fluorescence measurements to show that the popular mEos4b in its Green state populates two conformations (A and B), differing in side‐chain protonation of the conserved residues E212 and H62,  altering the hydrogen‐bond network in the chromophore pocket. The interconversion (protonation/deprotonation) between these two states, which occurs on the minutes time scale in the dark, becomes strongly accelerated in the presence of UV light, leading to a population shift. This work shows that the reversible photoswitching and Green‐to‐Red photoconversion properties differ between the A and B states. The chromophore in the A‐state photoswitches more efficiently and is proposed to be more prone to photoconversion, while the B‐state shows a higher level of photobleaching. Altogether, this data highlights the central role of conformational heterogeneity in fluorescent protein photochemistry.

## Graphical abstract

Solution NMR detects two distinct Green‐states of the photoconvertible fluorescent protein mEos4b with an altered hydrogen‐bonding network in the chromophore pocket. The relative population of these two conformational states is UV light dependent, providing a convenient way of assessing their different on‐to‐off photoswitching and Green‐to‐Red photoconversion properties.

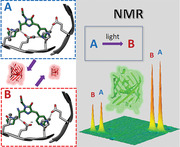

## 1 Introduction

**Figure 1:**
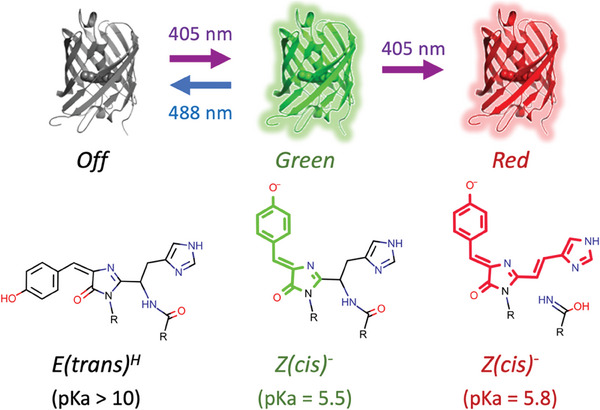
Phototransformations of Green mEos4b and corresponding chromophore structure and isomeric states. The relative population of protonated neutral and deprotonated anionic chromophore species can be derived from the chromophore pKa values given for each state.

Green‐to‐red photoconvertible fluorescent proteins (PCFPs) play a central role in advanced fluorescence microscopy approaches such as single molecule localization microscopy (SMLM).^[^ ^1^ ^]^ A number of such PCFPs have been derived from the tetrameric *Lobophyllia hemprichii* EosFP coral protein,^[^ ^2^ ^]^ such as the popular mEos3.2, mEos4b, PCStar, and mEosEM.^[^ ^3^, ^4^, ^5^, ^6^ ^]^ The mEos4b and mEosEM variants are particularly resistant to fixation conditions, including the conditions required for correlative light and electron microscopy.^[^ ^4^, ^6^ ^]^ mEos4b, like other PCFPs of anthozoan origin, bears a histidine as the first amino acid of the chromophore triad (His‐Tyr‐Gly) that autocatalytically maturates into a p‐HBI (4‐(p‐hydroxybenzylidine)−5‐imidazolinone) chromophore (Green‐state). Green‐to‐Red photoconversion then proceeds through absorption of a violet photon by the protonated neutral p‐HBI chromophore (**Figure** **1**). Photoconversion leads to backbone breakage at the CA atom linking residues F61 and H62 (mEos4b notation), resulting in increased chromophore electron conjugation and bathochromic shifts of the excitation and emission wavelengths.^[^ ^7^ ^]^ The Green‐state of mEos4b also shows substantial photochromism (Figure 1).^[^ ^8^ ^]^ Upon illumination with cyan light (488‐nm), mEos4b switches to a non‐fluorescent Off‐state, involving *cis‐to‐trans* isomerization and protonation of the chromophore. Contrary to photoconversion, on‐to‐off switching can be reversed by applying UV light (405 nm).

The photoconversion mechanism of Eos‐like PCFPs has been intensely discussed in the literature, but remains incompletely understood.^[^ ^7^, ^9^, ^10^, ^11^, ^12^, ^13^, ^14^ ^]^ Deciphering this mechanism is of key importance to better understand photoconversion kinetics and efficiency.^[^ ^15^, ^16^, ^17^ ^]^ A number of scenarios have been proposed, involving β‐elimination of type I or II,^[^ ^7^, ^10^, ^11^, ^12^, ^13^ ^]^ histidine rotamer adjustment,^[^ ^13^ ^]^ intersystem crossing to the triplet state,^[^ ^11^ ^]^ radical formation,^[^ ^14^ ^]^ and quadrupolar electrostatic coupling between neighboring residues.^[^ ^18^ ^]^ A consensus between these mechanisms is that the fully conserved E212 plays an essential role. Mutation of E212 to glutamine is known to abolish photoconversion.^[^ ^7^, ^19^ ^]^ However, the exact role of E212 along the photoconversion pathway remains questioned.

**Figure 2:**
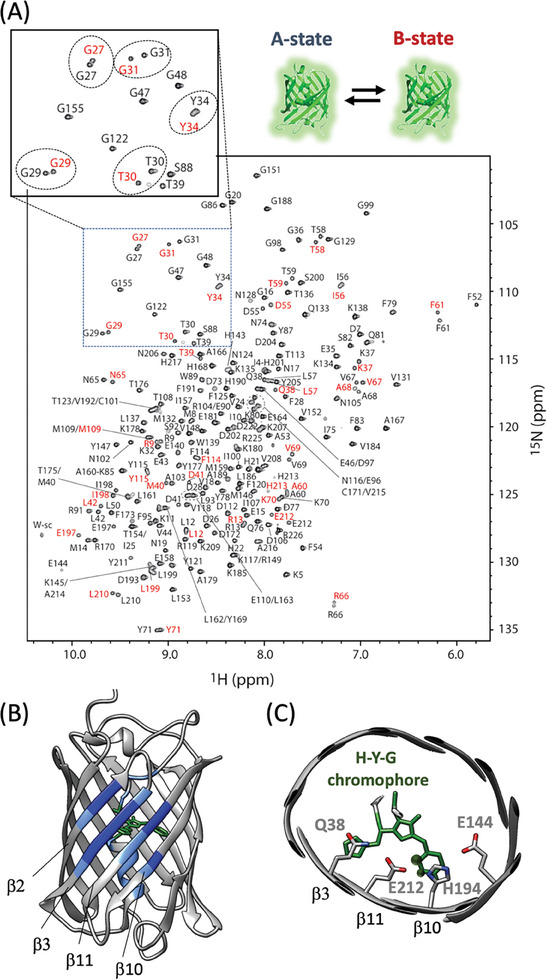
mEos4b Green‐state heterogeneity. A) ^1^H‐^15^N correlation spectrum recorded at 35 °C and pH 7.5. Cross peaks are annotated by their residue type and number. Well resolved B‐state peaks are highlighted in red. B) Large chemical shift changes between the A‐ and B‐states are color‐coded (dark blue: Δδ_HN_ > 1.0 ppm; light blue: 0.5 ppm < Δδ_HN_ < 1.0 ppm) on the crystal structure of mEos4b Green‐state (PDB 6GOY). C) Structural view of the chromophore in the mEos4b β‐barrel, and the Q38‐E212‐H194‐E144 motif. Chromophore ^1^H‐^13^C moieties that show NMR peak doubling are indicated by black balls.

In all known anthozoan PCFPs, E212 is part of a conserved Q38‐E212‐H194‐E144 amino‐acid motif, located directly beneath the chromophore (**Figure** **2**C). Residues in this motif form a hydrogen‐bond network that contributes to stabilize the chromophore in the protein scaffold. Intriguingly, E212 is observed to adopt an ill‐defined conformation in crystallographic structures of some PCFPs,^[^ ^3^, ^20^, ^21^, ^22^ ^]^ and in some cases, ensemble refinement procedures suggest a large set of conformations.^[^ ^8^ ^]^ This local heterogeneity of E212 might be fundamentally related to the enhanced flexibility of the chromophore pocket that seems to be necessary to catalyze photoconversion in PCFPs.^[^ ^23^ ^]^

Recently, solution NMR spectroscopy has demonstrated its potential for investigating the conformational dynamics of reversibly switchable fluorescent proteins,^[^ ^24^, ^25^, ^26^ ^]^ providing crucial information on the conformation and protonation states of the chromophore moiety and neighboring amino acid side chains.^[^ ^26^ ^]^ Here, we investigate by solution NMR and fluorescence approaches the conformational dynamics of mEos4b. We find that this PCFP exhibits two well‐defined conformational Green states that differ in terms of protonation and H‐bonding patterns of amino‐acid side chains in the chromophore pocket. We also study the exchange kinetics and thermodynamics of the two Green states in the dark and under UV illumination. Moreover, we discuss differences in the reversible photoswitching of both states, and conformational heterogeneities in the photoswitched Off‐state. Finally, we perform in situ NMR photoconversion experiments at different illumination power levels, which allow us to propose a photophysical model that accounts for the observed photoconversion kinetics.

## 2 Results and Discussion

### 2.1 Conformational Green‐State Heterogeneity of mEos4b

Despite the fact that X‐ray diffraction data of mEos4b crystals at cryogenic temperatures could be well fitted to a single protein conformation,^[^ ^27^ ^]^ solution NMR data showed a pronounced conformational heterogeneity of mEos4b in its Green state (Figure 2A). Sequential backbone NMR assignments revealed the presence of two distinct conformations, in the following termed A‐ and B‐states, that coexist with relative populations of 57 ± 2% (A‐state) and 43 ± 2% (B‐state) under the chosen experimental conditions. Interestingly, similar NMR spectral features are also observed for two other variants of the mEos family, PCStar, and the Dendra‐like^[^ ^22^ ^]^ mEos4b‐V69T mutant (Figure S1, Supporting Information), suggesting that the observed conformational Green‐state heterogeneity is a general feature of EosFP‐derived PCFPs.

Mapping the observed ^1^H, ^15^N chemical shift variations between the A‐ and B‐states on the structure of mEos4b (Figure 2B and Figure S2, Supporting Information) reveals the largest differences for residues in β‐strands β3, β10 and β11 facing the histidine and phenol moieties of the chromophore, and harboring the first 3 amino‐acid residues of the highly conserved and functionally important Q38‐E212‐H194‐E144 motif. Distinct CD_1_‐H and CD_2_‐H (as well as CE_1_‐H and CE_2_‐H) correlation peaks could be detected for the symmetry‐related C‐H sites of the chromophore's phenol moiety (Figure S3, Supporting Information), indicative of slow ring‐flip dynamics. Interestingly, only the ^13^C‐^1^H groups on one side of the phenol are sensitive to the A/B‐state heterogeneity, resulting in slightly different NMR signals that we could unambiguously assign to the phenol side pointing toward the side chain of E212 (Figure 2C and Figure S4A, Supporting Information). The A/B exchange kinetics is slow (*k*_ex_ < 1 s^−1^) at 35 °C as deduced from the observation of distinct NMR signals for A and B, and the absence of cross peaks detected in EXSY spectra.^[^ ^28^ ^]^

### 2.2 Coupled Protonation/Deprotonation Events in the Chromophore Pocket of mEos4b

**Figure 3:**
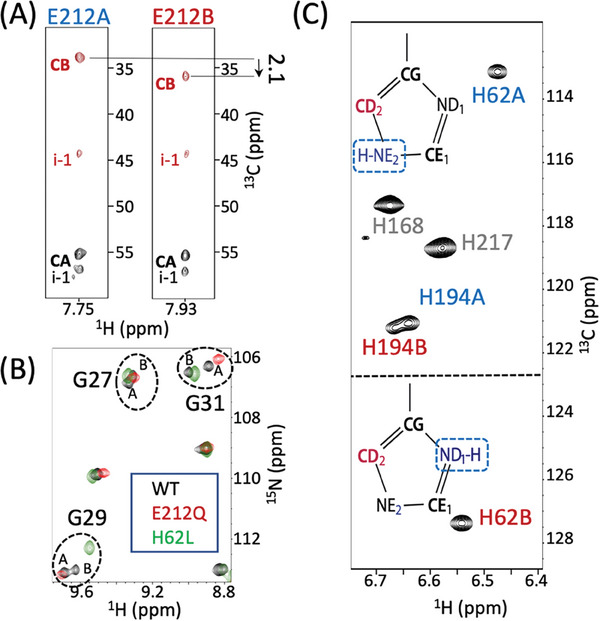
Protonation/deprotonation equilibria in Green mEos4b. A) Strips from a 3D Best‐TROSY HNCACB correlation spectrum,^[^ ^30^ ^]^ extracted at the ^15^N frequencies of E212 in the A‐ and B‐states. B) Spectral overlay (glycine region) of mEos4b WT (black), E212Q (red), and H62L (green) mutants. C) Tautomeric states of H62 and H194 side chains in the A‐ and B‐states, deduced from characteristic ^13^C chemical shifts at CD2 position.

In view of these initial NMR observations, and the fact that E212 is often not well defined in crystal structures of Eos‐like PCFPs,^[^ ^21^, ^22^ ^]^ we hypothesized that E212 protonation at its carboxylic side‐chain could be involved in the observed conformational heterogeneity. We therefore measured the ^13^CB chemical shifts of E212 in the A‐ and B‐states (**Figure** **3**A) as a reporter of its side chain protonation. The measured chemical shift difference of +2.1 ppm is in good agreement with reported values for glutamate deprotonation.^[^ ^28^ ^]^ In order to further validate the implication of E212 in the observed Green‐state heterogeneity, we performed additional NMR experiments on a mEos4b E212Q mutant that cannot undergo deprotonation at its side chain. Indeed, the recorded NMR spectra (Figure 3B) are in agreement with the presence of a single conformation, and ^13^C chemical shifts that closely match those of the wild‐type (WT) A‐state (Figure S5, Supporting Information). Therefore, we can conclude that the A‐state corresponds to a conformation with a protonated E212 (E212^OH^), while the B‐state is characterized by a negatively charged E212 (E212^O−^).

A‐B state heterogeneity was also detected for two histidine side chains, H194 that is part of the Q38‐E212‐H194‐E144 motif, as well as H62, the first residue of the chromophore‐forming tri‐peptide (Figure 3C). While only small chemical shift differences are detected for the ^13^CD_2_‐^1^H spin pair of H194, a 14‐ppm chemical shift difference is detected for the ^13^CD_2_ of H62 between the A‐ and B‐states. The ^13^C chemical shift of CD_2_ is particularly sensitive to the tautomeric state of the imidazole,^[^ ^28^, ^29^ ^]^ and the observed chemical shift values imply that H62 mainly populates the NE_2_‐H tautomer in the A‐state, while the ND_1_‐H tautomer is stabilized in the B‐state.

In order to investigate whether the presence of the H62 side chain is required for the observed partitioning of the E212 side‐chain protonation state, we prepared a mEos4b‐H62L mutant. No peak doubling, and thus no conformational heterogeneity, was observed in the NMR spectra of this Skylan‐NS like^[^ ^31^ ^]^ mutant (Figure 3B). Our NMR data thus point toward a coupled mechanism at the origin of the Green‐state heterogeneity, where the protonation/deprotonation of the E212 side chain carboxylate is accompanied by a tautomeric state change of the imidazole ring of H62, and vice versa. The protonation patterns can thus be described as E212^OH^‐H62^E2H^ (A‐state) and E212^O−^‐H62^D1H^ (B‐state).

### 2.3 Green mEos4b A‐ and B‐State Conformation and Dynamics

The chromophore's hydroxyphenyl moiety is deprotonated at ≈97% at pH ≥ 7.5, as deduced from the reported pKa and Hill coefficient,^[^ ^4^ ^]^ and confirmed by the absence of a pronounced absorbance band at 400 nm (Figure S6A, Supporting Information), and characteristic ^13^C chemical shifts of the hydroxyphenyl ring carbon CZ (Figure S4A, Supporting Information). NMR also provides information about chromophore rigidity, and in particular ring‐flip rates about the phenoxy bond of the chromophore's methine bridge.^[^ ^26^ ^]^ Such ring‐flip measurements for mEos4b at pH 8.5 and 35 °C reveal that the chromophore is structurally well stabilized in both A‐ and B‐state conformations, with ring‐flip rates *k*_RF_ < 1 s^−1^ (Figure S4B, Supporting Information).

**Figure 4:**
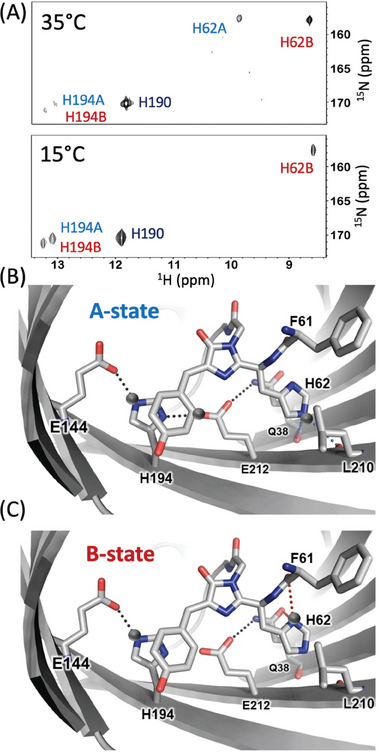
Hydrogen bonding patterns in Green mEos4b A‐ and B‐states plotted on the crystal structure (PDB 6GOY). A) ^1^H‐^15^N histidine SOFAST‐HMQC^[^ ^33^ ^]^ spectra of mEos4b recorded at 700 MHz and 15 °C (left), 35 °C (right). B,C) Potential H‐bonds (dashed lines) involving the Q38‐E212‐H194‐E144 motif and the H62 side chain in the A‐ and B‐states as inferred from the crystal structure and the NMR data. Protonation of the H62, H194, and E212 side chains is indicated by grey balls. A different view of these conformational states is provided in Figure S9, Supporting Information.

Additional structural information about the involvement of histidine side chains in hydrogen‐bond interactions is obtained from histidine ^1^H‐^15^N correlation spectra.^[^ ^32^ ^]^ At neutral pH, the labile nitrogen‐bound imidazole hydrogens only become NMR observable if they are protected from chemical exchange with the solvent by hydrogen bonding. For mEos4b, a total of 5 imidazole ^1^H‐^15^N correlation peaks could be detected (**Figure** **4**A) and unambiguously assigned to H62, H194, as well as His190 (Figure S7A, Supporting Information). Again, H62 and H194 give rise to distinct correlation peaks for the A‐ and B‐state conformations, revealing their engagement in hydrogen bonding interactions in both states.

The imidazole ^1^H‐^15^N correlation peaks detected for H62 and H194 show a distinctive temperature dependence. For H194, the weak peaks detected at 35 °C become significantly enhanced at 15 °C (Figure 4A). This suggests that H194 hydrogen‐bonding is stabilized at low temperature in both the A‐ and B‐states. The crystallographic structure indicates a H‐bond‐compatible short interatomic distance between the protonated imidazole nitrogen (NE_2_) of H194 and the carboxylic side chain group of E144 (R_O‐N_ = 2.8 Å). The most likely H‐bonding networks in this important Q38‐E212‐H194‐E144 motif are depicted in Figure 4B (and Figure S8A, Supporting Information), 4C (and Figure S8B, Supporting Information) for the A‐ and B‐states, respectively. Our NMR data, however, highlight that the H‐bond formed between E144 and H194 is highly dynamic at elevated temperature (35 °C). The presence of milliseconds time‐scale motions in the chromophore pocket of mEos4b is confirmed by extensive exchange line broadening observed for NMR signals of backbone amides located on strands β7 (142‐144), β10 (212‐213), and β11 (194‐196) comprising E144, H194, and E212. The situation is strikingly different for H62. In the A‐state, the ^1^H‐^15^N correlation peak detected at 35 °C (and 700 MHz ^1^H frequency) is about a factor of 3 weaker than the corresponding signal observed for the B‐state, despite the slightly higher A‐state population. The NMR intensity becomes further reduced at lower temperature, and the peak completely disappears (intensity below noise threshold) at T ≤ 15 °C. The magnetic field dependence of the NMR peak intensities (Figure S7B, Supporting Information) indicates an exchange process in the A‐state that is fast on the NMR chemical shift time scale *k*_ex_ ≳ 100 s^−1^ (ν_A_−ν_A’_). Exchange line broadening is also observed for the two ^1^H‐^13^C spin pairs in the imidazole ring of the H62 A‐state with a similar temperature and magnetic field dependence (Figure S7C, Supporting Information). Our NMR data are thus in agreement with a model where H62 in the A‐state exchanges between two (or more) H‐bonded conformations, while in the B‐state, hydrogen‐bonding of H62 is only little temperature dependent, indicative of a strongly H‐bonded conformation.

In the crystallographic structure, neither H62 NE_2_ (A‐state) nor ND_1_ (B‐state) is found at H‐bond‐compatible short interatomic distance to potential hydrogen acceptors. Thus, the low‐temperature X‐ray structure does not reflect the conformational side‐chain arrangements in the chromophore pocket of either the A‐ or B‐states, but rather some population‐weighted average. Figure 4B (and Figure S8A, Supporting Information), 4C (and Figure S8B, Supporting Information) show the most likely H‐bond patterns of H62 in the A‐ and B‐states, according to the crystal structure (R_O‐N_ distances ranging between 3.3 and 3.7 Å).

### 2.4 Conserved A/B‐State Heterogeneity in Photoswitched Off‐State

**Figure 5:**
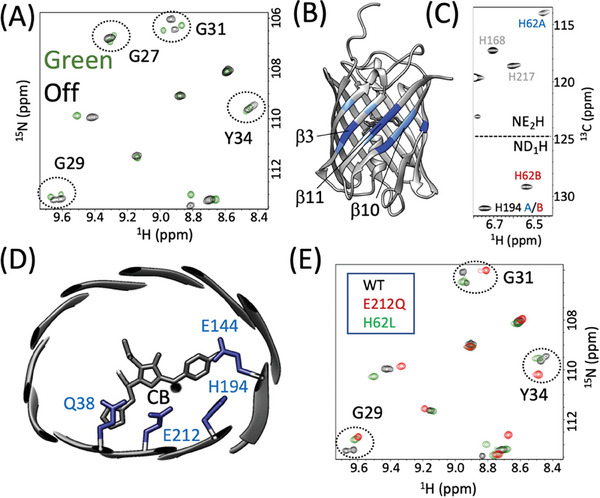
Off‐state conformational heterogeneity. A) Spectral overlay (glycine region) of mEos4b Green‐ (green) and Off‐state (black) ^1^H‐^15^N spectra (35 °C, pH 7.5). B) Large chemical shift differences between the A‐ and B‐states are color‐coded on the crystal structure of mEos4b Off‐state (PDB: 6GOZ). C) Tautomeric states of H62 and H194 side chains in the Off A‐ and B‐states, deduced from characteristic ^13^CD_2_ chemical shifts. D) Structural view of the E(trans) chromophore and Q38‐E212‐H194‐E144 motif in the Off‐state (left). The ^13^CB‐1H moiety (blue ball) of the chromophore shows peak doubling. E) Off‐state ^1^H‐^15^N spectra of mEos4b WT (black), E212Q (red), and H62L (green) mutants. The peak doubling observed for residues G29, G31, and Y34 in the WT is not observed in the two mutants.

Photoswitching of mEos4b involves a *Z(cis)* to *E(trans)* isomerization of the chromophore accompanied by protonation of the chromophore's tyrosine moiety (Figure 1).^[^ ^27^ ^]^ We therefore questioned whether the conformational Green‐state heterogeneity is conserved in the photoswitched Off‐state. NMR experiments of mEos4b under continuous 488‐nm illumination revealed the presence of two major conformational Off‐states (**Figure** **5**A). We could quantify the relative Off‐state populations to 45 ± 3% A and 55 ± 3% B. Plotting the observed amide ^1^H‐^15^N chemical shift differences between the Off‐A and Off‐B states points again to the β‐barrel region covering strands β3, β10, and β11 (Figure 5B and Figure S9, Supporting Information).

At the chromophore level, peak doubling is now observed for the ^1^H‐^13^C pair of the methine bridge CB‐site that in the crystal structure of the Off‐state is located close to the carboxylic side chain of E212 (Figure 5D and Figure S3, Supporting Information). A ^13^CB chemical shift difference of 1.3 ppm between the Off‐A and Off‐B states again suggests a change in protonation state of the E212 side chain. Also the tautomeric state difference of H62 between A and B is conserved in the Off‐state, while the imidazole side chain of H194 switches from a (NE_2_‐H)‐tautomer in the Green‐state to a (ND_1_‐H)‐tautomer in the Off‐state (Figure 5C). Similarly to our observations for the Green‐state, mutating either H62 or E212 removes this Off‐state conformational heterogeneity (Figure 5E).

### 2.5 UV Light Dependence of Green A/B‐State Equilibrium

**Figure 6:**
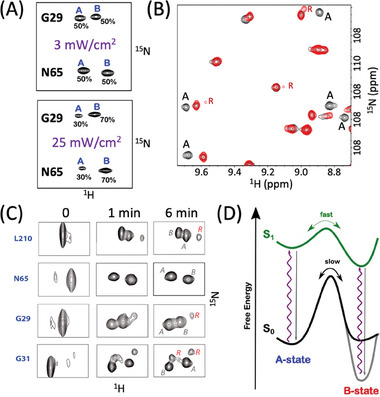
Free energy landscape of Green mEos4b. A/B‐state equilibrium at A) low‐power and B) high‐power (≈250 mW cm^−2^) UV (405 nm) illumination (red contours). C) A‐B exchange kinetics as experienced by 4 amide backbone sites in mEos4b after short (a few seconds) high power UV illumination. The first time point (0) has been recorded in a short acquisition time (1 min) resulting in apparent broader lines in the ^15^N dimension. Red‐state peaks that appear as a result of photoconversion under UV illumination are annotated (“R”). D) Schematic energy landscape of Green mEos4b in the ground (S_0_) and electronically excited (S_1_) states. The black and grey lines correspond to dark and UV‐light conditions, respectively.

Photoconversion of mEos4b from a Green to a Red fluorescent state requires UV light (Figure 1). We thus investigated the effect of UV illumination on the Green A‐ and B‐state populations. Under weak illumination conditions, where photoconversion kinetics are slow, we observed a partial conversion from the A‐ to the B‐state (**Figure** **6**A). At a power density of ≈25 mW cm^−2^ the equilibrium B‐state population has increased from 43% (in the dark) to about 70%. Increasing the UV power density by another order of magnitude (≈250 mW cm^−2^) results in only a slight further shift of the A/B‐state equilibrium to ≈80% B‐state (Figure 6B and Figure S10, Supporting Information). This finding suggests that even at the high power levels used in SMLM modalities, the A‐state remains populated to a significant extent.

This UV‐induced population shift allowed unambiguous identification of A‐ and B‐state peaks. Furthermore, it provided an opportunity to quantify the slow A/B exchange kinetics in the dark by off‐equilibrium real‐time 2D NMR. Figure 6C shows the thermal recovery of the A/B state population equilibrium at pH 7.5 and 35 °C after a short high‐power UV light pulse. These data fit to an A‐B exchange with a rate constant *k*_ex_ = 2 ± 1 10^−2^ s^−1^. Our observations imply that in the electronically excited S_1_ state, the B‐state conformation is energetically favored with respect to the A‐state. UV power densities of a few mW cm^−2^ are sufficient to create conditions where mEos4b molecules are efficiently shuffled from the A‐ to the B‐state (Figure 6D).

### 2.6 Green‐to‐Off Photoswitching

**Figure 7:**
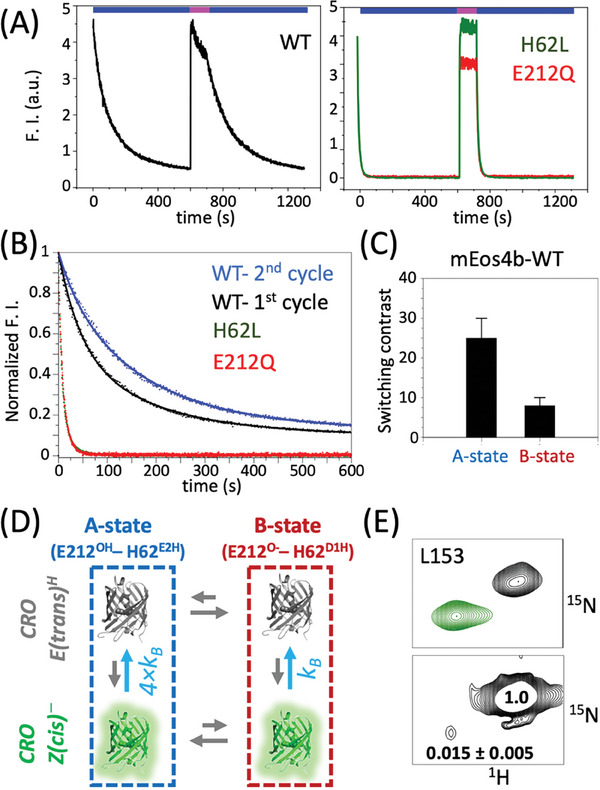
Photoswitching properties of mEos4b under 488‐nm illumination. A) Fluorescence intensity of mEos4b‐WT, E212Q and H62L mutants measured by applying a UV‐cyan light illumination scheme (indicated on top) B) Superposition of different switching cycles: WT first cycle (black), second cycle (blue); for E212Q (red) and H62L (green), the first 2 switching cycles are superposed. C) Apparent fluorescence switching contrast of WT A‐ and B‐states, obtained by global fitting the Green‐state decay (first and second) cycle assuming different A‐ and B‐state populations. D) On‐to‐off photoswitching model for mEos4b. E) NMR derived switching contrast, exemplified for the amide spin pair of residue L153. The upper spectrum shows a superposition of the Green‐ (green) and Off‐state (black) spectra, while the lower graph shows the same Off‐state spectrum plotted at low contour levels in order to observe the residual Green‐state peak.

The ability to change the relative A/B‐state population ratio by UV light enabled us to investigate how the conformational heterogeneity influences the photophysics of mEos4b. Ensemble fluorescence measurements using an alternation of cyan (488 nm) and violet (405 nm) light was used to check for differences in photoswitching kinetics and contrast between the A‐ and B‐states (**Figure** **7**A). The first switching cycle starts from thermodynamic equilibrium, with an A/B state population ratio (≈57%/43%), while subsequent cycles are representative of the switching behavior of an A/B‐state population ratio (≈30%/70%). The UV power was adjusted to allow a significant A‐to‐B population shift and effective off‐to‐on back switching, but limited Green‐to‐Red photoconversion. Interestingly, faster fluorescence intensity decay is observed for the first cycle compared to cycles recorded after an UV light pulse (Figure 7B). Adding a thermal relaxation delay makes the system again behave similar to the first cycle (Figure S11, Supporting Information). We ascribe this different switching behavior to the UV‐induced change in A/B population ratio. Fitting our kinetic data to a bi‐exponential model, accounting for 2 independently switching states (A and B) with different kinetics and switching contrast, revealed that photoswitching in the A‐state is about four times faster than in the B‐state (Figure 7D). We also measured photo‐switching kinetics for the mEos4b E212Q and H62L mutants that both populate a unique conformation which, in the case of E212Q, resembles the WT A‐state (Figure 7A,B). In both cases, photoswitching is 4–5 times faster than the A‐state of mEos4b‐WT, and no difference between switching cycles is observed in agreement with our assumption that this difference is mainly caused by the UV light‐induced changes of the A/B‐state equilibrium.

Our data fitting also provides a measure of the apparent switching contrast for the A‐ and B‐states. The faster switching A‐state shows a higher apparent switching contrast (lower residual Off‐state fluorescence) than the slower switching B‐state (Figure 7C). The correlation between faster switching kinetics and higher contrast, previously observed in several RSFPs,^[^ ^8^, ^34^, ^35^ ^]^ also holds true for the two mEos4b mutants E212Q and H62L that both have an apparent switching contrast of more than 200. Our observation of a moderate switching contrast (< 30) of both the A‐ and B‐states of mEos4b, as deduced from fluorescence measurements, is not fully in line with the residual Green‐state population detected in the Off‐state NMR spectra recorded under 448‐nm illumination (Figure 7E). An apparent NMR switching contrast of 65 ± 15 is obtained from the average (and standard deviation) of intensity ratios measured for 30 well resolved Green‐state signals that do not overlap with any Off‐state peak. This value is more than a factor of 2 higher than the switching contrast derived from fluorescence measurements (Figure 7C). Residual Off‐state fluorescence is generally ascribed to an incomplete conformational transition from a fluorescent *Z(cis)^−^* to a non‐fluorescent *E(trans)^Η^* chromophore configuration due to residual on‐switching by the off‐switching cyan light. Moreover, fluorescence measurements revealed a *E(trans)* chromophore pKa > 10 in mEos4b (Figure S12, Supporting Information), in line with previous reports,^[^ ^36^ ^]^ suggesting an insignificant amount of fluorescent anionic *E(trans)* chromophores at pH 7.5. Our NMR data thus show that additional factors influence the observed switching contrast in fluorescence measurements.

### 2.7 Green‐to‐Red Photoconversion

We further investigated by NMR Green‐to‐Red photoconversion at low UV illumination power (≈2.5 to 25 mW cm^−2^). Under these conditions the A/B state population ratio can be tuned by the applied UV power as demonstrated above (Figure 6A), and photoconversion occurs on timescales of minutes to hours, that are compatible with real‐time 2D NMR data acquisition.

**Figure 8:**
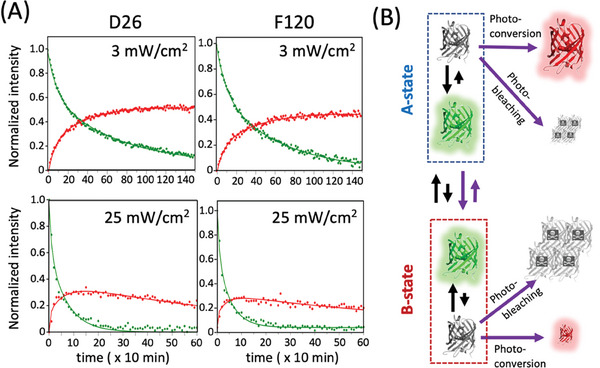
mEos4b Green‐to‐Red photoconversion. A) NMR real time data recorded under continuous UV (405‐nm) illumination at different light power densities. Green state decay and Red‐state build‐up curves are plotted for residues D26 and F120. The NMR‐derived photoconversion efficiencies are ≈50% at 3 mW cm^−2^ and ≈30% at 25 mW cm^−2^. B) Simple photoconversion model that accounts for the NMR kinetics data.

Many residues in mEos4b experience distinct ^1^H‐^15^N chemical shifts in the Green‐ and Red‐states (Figure S13, Supporting Information) thus allowing to follow NMR peak intensity changes simultaneously for the Green‐ and Red‐states during photoconversion. **Figure** **8**A shows Green‐state decay and Red‐state build‐up curves for selected ^1^H‐^15^N pairs that show no peak doubling and give rise to well‐resolved ^1^H‐^15^N correlation peaks in both the Green‐ and Red‐state NMR spectra. The plotted data have been recorded with UV power densities of 3 (top panel) and 25 mW cm^−2^ (bottom panel), measured at the sample top. The kinetic data extracted for a total of 6 mEos4b residues, comprising sites with distinct Green A‐ and B‐state signatures, are well fitted to a biphasic kinetic model, assuming identical rate constants for Green‐state decay and Red‐state build‐up, and relative amplitudes of 0.35 ± 0.05 for the fast phase and 0.65 ± 0.05 for the slow phase (Figure S14, Supporting Information). Undistinguishable Green‐state decay kinetics are observed for the A‐ and B‐states, in agreement with fast A‐B exchange kinetics compared to the photoconversion time scale. Both (fast and slow) kinetic rates increase linearly with the applied UV power density, indicating that the involved photochemical transformations are single‐photon processes.

Apparent photoconversion efficiencies are obtained from the ratio between the maximal Red‐state peak intensity reached during the kinetics and the initial Green‐state intensity. As can be appreciated from Figure 8A, the photoconversion efficiency reaches up to ≈50% at very low UV power density (3 mW cm^−2^), but decreases to ≤30% at 25 mW cm^−2^. Assuming that Red‐state photobleaching is also a single‐photon process (in agreement with our NMR data), this observation suggests that the difference in the Green A/B‐state population equilibrium may be at the origin of the observed drop in photoconversion efficiency with increasing UV power density. The observation that a higher A‐state population correlates with an improved photoconversion efficiency suggests a photophysical model where photoconversion takes place predominantly in the A state, while Green‐state photobleaching is more pronounced in the B state (Figure 8B). This simple model allows to reproduce the main features of our NMR data using SMIS^[^ ^37^ ^]^ simulations (Figure S15 and Table S1, Supporting Information),

The NMR‐derived photoconversion efficiencies of mEos4b are lower than what has been reported in the literature based on measurements at the single‐molecule level.^[^ ^38^ ^]^ This discrepancy may possibly be explained by the presence of strong additional illumination at 561 nm in optical microscopy experiments, resulting in enhanced photoconversion efficiencies.^[^ ^39^ ^]^ We thus cannot exclude that photoconversion of mEos4b is based on a more complex scenario than the simple model proposed in Figure 8B, involving additional photo‐induced transformations and states. Future NMR experiments in the presence of both illumination lasers will allow to address this issue.

### 2.8 Implications for the Photoconversion Mechanism(s) of mEos4b

Several mechanistic scenarios proposed in the literature assume as the starting point for photoconversion a conformation with H194 in an imidazolium cationic form.^[^ ^11^, ^12^, ^13^, ^18^ ^]^ Our NMR data clearly show that H194 adopts a neutral tautomeric form in the Green‐state, strongly suggesting that photoconversion to the Red‐state occurs in this form. Previous work by the Watcher group on the Least Evolved Ancestor (LEA) PCFP^[^ ^18^ ^]^ emphasizes that a flexible chromophore environment is required for photoconversion, particularly at the level of H62 which likely needs to undergo conformational changes during the photoconversion process. Our data are fully consistent with this notion of a high conformational plasticity of the chromophore environment in the Green A state. Although NMR does not provide direct evidence of the photochemical mechanism at play, a photoconversion model similar to the one introduced by Kim et al.^[^ ^13^ ^]^ and starting from the Green A‐state, is in good agreement with our NMR data (Figure S16, Supporting Information).^[^ ^13^ ^]^ This model proposes an excited state proton transfer (ESPT) from the protonated E212 to H62‐ND_1_ after transient rotamer adjustment of H62 bringing the E212 carboxylate and H62 imidazole in close proximity. Photoconversion then proceeds via concerted β‐elimination involving backward proton shuttling from H62‐CB to E212.

If photoconversion also occurs from the Green B‐state, different scenarios are possible. First, a mechanism similar to that proposed by Fare et al.,^[^ ^14^ ^]^ requiring E212 to act as a base and involving rapid formation of the ethylenic CA═CB bond on H62 followed by slow decay of a cationic imidazole radical, could constitute a plausible scenario. In the B‐state, H62 is singly protonated at its ND_1_ and is thus poised for excited state proton transfer to F61. Hence, the mechanism proposed by Lelimousin et al.^[^ ^11^ ^]^ may also be possible, involving the triplet state and not requiring any proton shuttling from E212. Such a mechanism could in fact relate to so‐called primed photoconversion in Dendra2 variants,^[^ ^40^, ^41^ ^]^ in which E212 maintains a salt bridge with R66 and may thus be predominantly deprotonated as observed for the B state.

## 3 Conclusion

PCFPs are an essential tool for super‐resolution fluorescence microscopy. A better understanding of the photophysical and photochemical pathways underlying photoconversion and photoswitching is essential for future efforts aiming at engineering improved PCFP variants for particular applications. Most of the mechanistic knowledge reported in the literature has been derived from crystallographic structures and quantum‐chemical calculations. Here, we have demonstrated that solution NMR spectroscopy provides critical additional information on conformational dynamics and structural heterogeneity in the chromophore pocket of PCFPs from the Eos family. Two conformations of similar free energy coexist in solution, and their populations (free energies) and exchange kinetics (free energy barriers) are modulated by UV light. Furthermore, we could establish that the two NMR‐observed Green states correspond to different local hydrogen‐bond networks caused by a concerted change in the protonation state at the side chains of H62 and E212. Finally, combining NMR and fluorescence data we could show how these two states differ in terms of their photoswitching and photoconversion behavior, and shed some new light on the underlying mechanisms.

The faster photoswitching kinetics of the Green A‐state relative to the B‐state should be taken into account when EosFP‐derived RSFPs are employed in applications exploiting photoswitching rates quantitatively.^[^ ^42^ ^]^ In PALM applications, our hypothesis that photoconversion is more efficient starting from the Green A‐state would suggest the use of low‐UV illumination schemes to minimize B‐state occupancy and maximize photoconversion efficiency. However, such a strategy might be challenged by practical considerations (e.g., PALM data collection time) and additional complexity in PCFP's photophysics such as photobleaching by readout light.^[^ ^43^ ^]^ The possibility to engineer mEos4b variants experiencing a population shift toward the Green A‐state offers an interesting perspective. However, the fundamental question of whether photoconversion capability and A/B‐state heterogeneity are intimately coupled should be addressed first.

In summary, we have shown that adding solution NMR spectroscopy with in situ sample illumination capabilities to the panoply of biophysical techniques used for the characterization of fluorescent protein markers improves our understanding of their photophysical and photochemical properties, as required for rational design and engineering of improved fluorescent protein variants.

## Conflict of Interest

The authors declare no conflict of interest.

## Supporting information

Supporting Information

## Data Availability

The data that support the findings of this study are available from the corresponding author upon reasonable request.

## References

[1] D. M. Shcherbakova , P. Sengupta , J. Lippincott‐Schwartz , V. V. Verkhusha , Annu. Rev. Biophys. 2014, 43, 303. 24895855 10.1146/annurev-biophys-051013-022836 PMC4254894

[2] J. Wiedenmann , S. Ivanchenko , F. Oswald , F. Schmitt , C. Röcker , A. Salih , K.‐D. Spindler , G. U. Nienhaus , Proc. Natl. Acad. Sci. USA 2004, 101, 15905. 15505211 10.1073/pnas.0403668101 PMC528746

[3] M. Zhang , H. Chang , Y. Zhang , J. Yu , L. Wu , W. Ji , J. Chen , B. Liu , J. Lu , Y. Liu , J. Zhang , P. Xu , T. Xu , Nat. Methods 2012, 9, 727. 22581370 10.1038/nmeth.2021

[4] M. G. Paez‐Segala , M. G. Sun , G. Shtengel , S. Viswanathan , M. A. Baird , J. J. Macklin , R. Patel , J. R. Allen , E. S. Howe , G. Piszczek , H. F. Hess , M. W. Davidson , Y. Wang , L. L. Looger , Nat. Methods 2015, 12, 215. 25581799 10.1038/nmeth.3225 PMC4344411

[5] M. Zhang , Z. Fu , C. Li , A. Liu , D. Peng , F. Xue , W. He , S. Gao , F. Xu , D. Xu , L. Yuan , F. Zhang , Z. Xu , T. Xu , P. Xu , Nano Lett. 2020, 20, 2197. 31576756 10.1021/acs.nanolett.9b02855

[6] Z. Fu , D. Peng , M. Zhang , F. Xue , R. Zhang , W. He , T. Xu , P. Xu , Nat. Methods 2020, 17, 55. 31611693 10.1038/s41592-019-0613-6

[7] K. Nienhaus , G. U. Nienhaus , J. Wiedenmann , H. Nar , Proc. Natl. Acad. Sci. USA 2005, 102, 9156. 15964985 10.1073/pnas.0501874102 PMC1166600

[8] E. De Zitter , J. Ridard , D. Thédié , V. Adam , B. Lévy , M. Byrdin , G. Gotthard , L. Van Meervelt , P. Dedecker , I. Demachy , D. Bourgeois , J. Am. Chem. Soc. 2020, 142, 10978. 32463688 10.1021/jacs.0c01880

[9] H. Mizuno , T. K. Mal , K. I. Tong , R. Ando , T. Furuta , M. Ikura , A. Miyawaki , Mol. Cell 2003, 12, 1051. 14580354 10.1016/s1097-2765(03)00393-9

[10] H. Tsutsui , H. Shimizu , H. Mizuno , N. Nukina , T. Furuta , A. Miyawaki , Chem. Biol. 2009, 16, 1140. 19942137 10.1016/j.chembiol.2009.10.010

[11] M. Lelimousin , V. Adam , G. U. Nienhaus , D. Bourgeois , M. J. Field , J. Am. Chem. Soc. 2009, 131, 16814. 19886627 10.1021/ja905380y

[12] X. Li , L. W. Chung , H. Mizuno , A. Miyawaki , K. Morokuma , J. Phys. Chem. B 2010, 114, 16666. 21082854 10.1021/jp1101779

[13] H. Kim , T. Zou , C. Modi , K. Dörner , T. J. Grunkemeyer , L. Chen , R. Fromme , M. V. Matz , S. B. Ozkan , R. M. Wachter , Structure 2015, 23, 34. 25565105 10.1016/j.str.2014.11.011 PMC4370283

[14] C. Fare , L. Yuan , V. Cordon‐Preciado , J. J. Michels , M. J. Bearpark , P. Rich , J. J. Van Thor , J. Phys. Chem. B 2020, 124, 7765. 32805110 10.1021/acs.jpcb.0c04587

[15] S. Wang , J. R. Moffitt , G. T. Dempsey , X. S. Xie , X. Zhuang , Proc. Natl. Acad. Sci. USA 2014, 111, 8452. 24912163 10.1073/pnas.1406593111 PMC4060684

[16] D. Thédié , R. Berardozzi , V. Adam , D. Bourgeois , J. Phys. Chem. Lett. 2017, 8, 4424. 28850784 10.1021/acs.jpclett.7b01701

[17] J. Wulffele , D. Thédié , O. Glushonkov , D. Bourgeois , J. Phys. Chem. Lett. 2022, 13, 5075. 35653150 10.1021/acs.jpclett.2c00933

[18] H. Kim , T. J. Grunkemeyer , C. Modi , L. Chen , R. Fromme , M. V. Matz , R. M. Wachter , Biochemistry 2013, 52, 8048. 24134825 10.1021/bi401000e

[19] G. U. Nienhaus , K. Nienhaus , A. Hölzle , S. Ivanchenko , F. Renzi , F. Oswald , M. Wolff , F. Schmitt , C. Röcker , B. Vallone , W. Weidemann , R. Heilker , H. Nar , J. Wiedenmann , Photochem. Photobiol. 2006, 82, 351. 16613485 10.1562/2005-05-19-RA-533

[20] V. Adam , K. Nienhaus , D. Bourgeois , G. U. Nienhaus , Biochemistry 2009, 48, 4905. 19371086 10.1021/bi900383a

[21] J.‐P. Colletier , M. Sliwa , F.‐X. Gallat , M. Sugahara , V. Guillon , G. Schirò , N. Coquelle , J. Woodhouse , L. Roux , G. Gotthard , A. Royant , L. M. Uriarte , C. Ruckebusch , Y. Joti , M. Byrdin , E. Mizohata , E. Nango , T. Tanaka , K. Tono , M. Yabashi , V. Adam , M. Cammarata , I. Schlichting , D. Bourgeois , M. Weik , J. Phys. Chem. Lett. 2016, 7, 882. 26866390 10.1021/acs.jpclett.5b02789

[22] R. Berardozzi , V. Adam , A. Martins , D. Bourgeois , J. Am. Chem. Soc. 2016, 138, 558. 26675944 10.1021/jacs.5b09923

[23] R. Wachter , Mol. Sci. 2017, 18, 1792.

[24] H. Mizuno , T. K. Mal , M. Wälchli , A. Kikuchi , T. Fukano , R. Ando , J. Jeyakanthan , J. Taka , Y. Shiro , M. Ikura , A. Miyawaki , Proc. Natl. Acad. Sci. USA 2008, 105, 9227. 18574155 10.1073/pnas.0709599105 PMC2453726

[25] N.‐E. Christou , I. Ayala , K. Giandoreggio‐Barranco , M. Byrdin , V. Adam , D. Bourgeois , B. Brutscher , Biophys. J. 2019, 117, 2087. 31733726 10.1016/j.bpj.2019.10.035 PMC6895687

[26] N. E. Christou , K. Giandoreggio‐Barranco , I. Ayala , O. Glushonkov , V. Adam , D. Bourgeois , B. Brutscher , J. Am. Chem. Soc. 2021, 143, 7521. 33966387 10.1021/jacs.1c02442

[27] E. De Zitter , D. Thédié , V. Mönkemöller , S. Hugelier , J. Beaudouin , V. Adam , M. Byrdin , L. Van Meervelt , P. Dedecker , D. Bourgeois , Nat. Methods 2019, 16, 707. 31285624 10.1038/s41592-019-0462-3

[28] G. Platzer , M. Okon , L. P. Mcintosh , J. Biomol. NMR 2014, 60, 109. 25239571 10.1007/s10858-014-9862-y

[29] J. A. Vila , Y. A. Arnautova , Y. Vorobjev , H. A. Scheraga , Proc. Natl. Acad. Sci. USA 2011, 108, 5602. 21422292 10.1073/pnas.1102373108 PMC3078339

[30] Z. Solyom , M. Schwarten , L. Geist , R. Konrat , D. Willbold , B. Brutscher , J. Biomol. NMR 2013, 55, 311. 23435576 10.1007/s10858-013-9715-0

[31] X. Zhang , M. Zhang , D. Li , W. He , J. Peng , E. Betzig , P. Xu , Proc. Natl. Acad. Sci. USA 2016, 113, 10364. 27562163 10.1073/pnas.1611038113 PMC5027434

[32] N. E. Christou , B. Brutscher , J. Biomol. NMR 2018, 72, 115. 30465113 10.1007/s10858-018-0216-z

[33] P. Schanda , B. Brutscher , J. Am. Chem. Soc. 2005, 127, 8014. 15926816 10.1021/ja051306e

[34] V. Adam , K. Hadjidemetriou , N. Jensen , R. L. Shoeman , J. Woodhouse , A. Aquila , A.‐S. Banneville , T. R. M. Barends , V. Bezchastnov , S. Boutet , M. Byrdin , M. Cammarata , S. Carbajo , N. E. Christou , N. Coquelle , E. De La Mora , M. El Khatib , T. M. Chicano , R. B. Doak , F. Fieschi , L. Foucar , O. Glushonkov , A. Gorel , M. L. Grünbein , M. Hilpert , M. Hunter , M. Kloos , J. E. Koglin , T. J. Lane , M. Liang , et al., ChemPhysChem 2022, 23, 202200192. 10.1002/cphc.202200192 PMC9804819 35959919

[35] S. Duwe , E. De Zitter , V. Gielen , B. Moeyaert , W. Vandenberg , T. Grotjohann , K. Clays , S. Jakobs , L. Van Meervelt , P. Dedecker , ACS Nano 2015, 9, 9528. 26308583 10.1021/acsnano.5b04129

[36] S. Gayda , K. Nienhaus , G. U. Nienhaus , Biophys. J. 2012, 103, 2521. 23260054 10.1016/j.bpj.2012.11.011 PMC3525849

[37] D. Bourgeois , Commun. Biol. 2023, 6, 53. 36646743 10.1038/s42003-023-04432-x PMC9842740

[38] N. Durisic , L. Laparra‐Cuervo , Á. Sandoval‐Álvarez , J. S. Borbely , M. Lakadamyali , Nat. Methods 2014, 11, 156. 24390439 10.1038/nmeth.2784

[39] M. Sun , K. Hu , J. Bewersdorf , T. D. Pollard , Biophys. J. 2021, 120, 21. 33217381 10.1016/j.bpj.2020.11.006 PMC7820738

[40] M. A. Mohr , A. Y. Kobitski , L. R. Sabater , K. Nienhaus , C. J. Obara , J. Lippincott‐Schwartz , G. U. Nienhaus , P. Pantazis , Angew. Chem., Int. Ed. 2017, 56, 11628. 10.1002/anie.201706121 28661566

[41] B. Turkowyd , A. Balinovic , D. Virant , H. G. G. Carnero , F. Caldana , M. Endesfelder , D. Bourgeois , U. Endesfelder , Angew. Chem., Int. Ed. 2017, 56, 11634. 10.1002/anie.201702870 28574633

[42] R. Chouket , A. Pellissier‐Tanon , A. Lahlou , R. Zhang , D. Kim , M.‐A. Plamont , M. Zhang , X. Zhang , P. Xu , N. Desprat , D. Bourgeois , A. Espagne , A. Lemarchand , T. L. Saux , L. Jullien , Nat. Commun. 2022, 13, 1482. 35304491 10.1038/s41467-022-29172-0 PMC8933551

[43] A. Acharya , A. M. Bogdanov , B. L. Grigorenko , K. B. Bravaya , A. V. Nemukhin , K. A. Lukyanov , A. I. Krylov , Chem. Rev. 2016, 117, 758. 27754659 10.1021/acs.chemrev.6b00238

