# Supplementary material for: Structural Heterogeneity in a Phototransformable Fluorescent Protein Impacts its Photochemical Properties
Source: Adv Sci (Weinh). 2023 Dec 25;11(10):2306272. doi: 10.1002/advs.202306272 (PMC10933604; doi:10.1002/advs.202306272)
Supplement: Supplementary file 1 — Supporting Information [file ADVS-11-2306272-s001.pdf]

ADVANCED SCIENCE
Open Access

Supporting Information

for *Adv. Sci.*, DOI 10.1002/advs.202306272

Structural Heterogeneity in a Phototransformable Fluorescent Protein Impacts its Photochemical Properties

Arijit Maity, Jip Wulffelé, Isabel Ayala, Adrien Favier, Virgile Adam, Dominique Bourgeois and Bernhard Brutscher**

Supporting Information

Structural Heterogeneity in a Phototransformable Fluorescent Protein impacts its Photochemical Properties

Arijit Maity, Jip Wulffelé, Isabel Ayala, Adrien Favier, Virgile Adam, Dominique Bourgeois, and Bernhard Brutscher**

Table of Contents

EXPERIMENTAL PROCEDURES 3

NMR sample preparation *3*

NMR experiments *3*

Sample preparation for ensemble fluorescence microscopy *5*

Ensemble fluorescence microscopy experiments *6*

pKa measurement of the switched-off state *6*

Photophysical simulations *6*

SUPPLEMENTARY TABLE S1 8

SUPPLEMENTARY FIGURES 9

***Figure S1**: PCFPs derived from Lobophyllia hemprichii EosFP* *9*

***Figure S2:** Green A-B-state chemical shift differences* *10*

***Figure S3:** NMR signature of p-HBI chromophore* *11*

***Figure S4:** Chromophore NMR resonance assignment and ring flips* *12*

***Figure S5:** NMR chemical shift differences between WT A- B-states and E212Q mutant* *13*

***Figure S6** : UV-Vis absorbance spectra* *14*

***Figure S7:** Histidine H-bonding and dynamics in mEos4b-WT* *15*

***Figure S8**: Hydrogen bonding in Green A- and B-states* *16*

***Figure S9:** Off A-B-state chemical shift differences* *17*

***Figure S10**: Green mEos4B NMR spectra with/without UV illumination* *18*

***Figure S11:** Fluorescence data of mEos4b photoswitching* *19*

***Figure S12:** pKa measurement of photoswitched Off state* *20*

***Figure S13**: NMR spectrum of Red mEos4B* *21*

***Figure S14**: NMR photoconversion kinetics* *22*

***Figure S15**: SMIS simulations of Green-to-Red photoconversion* *28*

***Figure S16:** Proposed main photoconversion model* *29*

REFERENCES 30

EXPERIMENTAL PROCEDURES

NMR sample preparation

Wild type (WT) mEos4b and mutants were cloned into the kanamycine-resistant expression plasmid pET28a allowing protein over-expression with a N-terminus 6-residue His tag. *Escherichia coli* BL21(DE3) cells were transformed with a plasmid carrying the target protein. Cells were adapted from rich LB medium to minimal M9 medium in two steps over 24h. M9 medium with isotopically enriched ^{15}N NH_4Cl (1 g/L) and ^{13}C-glucose (2 g/L) was inoculated with adapted cells, and grown at 37°C until the cultures reached an OD (600 nm) of 0.7. Then, protein over-expression was induced by adding 1mM IPTG (isopropyl b-D-thiogalactopyranoside) and induction was performed during 4 hours at 37°C. After induction, cells were harvested by centrifugation and lysed by sonication in the following buffer: 20 mM HEPES pH 7.5, 150 mM NaCl), supplemented with a cOmplete^{TM}EDTA-Free (Roche) tablet. After sonication the lysate was clarified by centrifugation at 46000g during 40 min. The supernatant was then loaded on a Ni-NTA (Qiagen) column pre-equilibrated with the same buffer. Non-specifically bound protein was removed by an additional washing step containing 25 mM imidazole. Protein elution was performed using a high concentration of imidazole, and a last step of purification was made using a Size Exclusion Chromatography (SEC) S75 (Cytiva) column equilibrated in 20 mM HEPES pH 7.5, 150 mM NaCl Buffer. Elution fractions containing the protein were dialyzed against the NMR buffer (50 mM HEPES, pH 7.5).

NMR samples were prepared by adding 100-200 μM of protein to 300 μL of buffer solution containing 5% (v/v) D_2O, filled into 5 mm Shigemi NMR tubes. The samples were kept at 4°C between NMR experiments

NMR experiments

NMR experiments were performed on Bruker Avance III-HD spectrometers (Billerica, MA), operating at magnetic field strengths (^1H Larmor frequencies) of 700, 850, or 950 MHz and equipped with cryogenically cooled triple-resonance probes and pulsed z-field gradients. NMR measurements were performed at 35°C, except for experiments probing the T-dependence of NMR observables. NMR data processing was performed using TopSpin 3.5 (Bruker BioSpin), while data analysis was done using the CCPNMR Analysis 2 software.[1]

2D NMR: Amide ^{1}H-^{15}N correlation spectra were recorded using a BEST-TROSY pulse scheme [2] with the ^{15}N carrier centered at 120 ppm, and the ^{1}H band selective pulses covering a band width of 8.7 ± 2.5 ppm. Histidine ^{1}H-^{15}N side-chain correlation experiments were based on a SOFAST-HMQC [3,4] pulse sequence with the ^{15}N carrier centered at 160 ppm, and the ^{1}H band selective pulses covering a band width of 11 ± 2.5 ppm. For aromatic side chain ^{1}H-^{13}C moieties, CT-BEST-HSQC or standard BEST-HSQC experiments[5] were performed with typical acquisition times of 70 ms (^{1}H), 14 ms (^{13}C-CT), and 7 ms (^{13}C- non CT).

NMR assignments: For sequential assignment of mEos4b in the Green- and Off-states, a set of 6 3D BEST-TROSY-HNC spectra [6] were recorded: HNCO, HNcoCA, HNCA, HNcaCO, HNCACB, and HNcoCACB. Semi-automated resonance assignment was realized with the CCPNMR Analysis 2 software. Specific assignments to either the A- or B-states were achieved by distinct correlation patterns (differences in CA, CO, and CB frequencies). In case of the Green state(s), these assignments could be independently confirmed (or evtl. corrected) by recording ^{1}H-^{15}N correlation spectra under high-power UV illumination that shifts the population equilibrium towards the B-state, or at lower pH where the A-state becomes highly populated. Such an independent assignment validation was not possible for the Off A- and B-states, thus leaving some ambiguities for distinguishing A- and B-state signals in the photoswitched Off state.

NMR chemical shifts (^{1}HN, ^{15}N, ^{13}CO, ^{13}CA, and ^{13}CB) measured for mEos4b (pH 7.5, 35°C) in the Green and Off states have been deposited with the BMRB under accession number 51879. Additional side chain resonance assignment for aromatic residues was achieved from a series of BEST-HCC, BEST-HNC correlation experiments,[5] as well as 2D ^{1}H-^{1}H NOESY.

NMR experiments for dynamics and kinetics: (i) Chromophore ring flip rates were estimated through ^{1}H-^{13}C EXSY-type experiments. The pulse sequence used is based on the aromatic BEST-HSQC experiment,[5] with an additional delay T inserted after the initial ^{1}H-^{13}C INEPT transfer step. During this exchange delay T, the spin system is in a $2C_ZH_Z$ spin state. (ii) Real-time kinetics measurements, e.g. thermal Off-to-Green relaxation or Green-to-Red photoconversion, were measured by recording a series of 2D ^{1}H-^{15}N BEST-TROSY correlation spectra during the kinetic event. The acquisition time of a single 2D data set was typically adjusted to about 10 minutes. Kinetic traces were then extracted from such pseudo-3D data sets as NMR peak intensities measured as a function of time, either for individual sites (residues),

or summed over a series of peaks (showing the same kinetics) to enhance the signal-to-noise ratio.

All NMR experiments used in this study are implemented in the NMRlib pulse sequence library[7] for Bruker spectrometers that can be freely downloaded from the IBS web site (https://www.ibs.fr/research/scientific-output/software/pulse-sequence-tools/).

In-situ NMR sample illumination was achieved using a setup described recently.[8] In short a combiner box houses the 2 laser sources used in this study (488 nm and 405 nm) that are combined into a single optical output which is connected via an optical multimode fiber to the NMR sample tube (Shigemi plunger).The maximum power output for each laser is on the order of 150 mW. This in-situ illumination device is portable and can thus be connected to any of the 3 NMR spectrometers used for this study.

Quantification of state populations: Quantitative information about relative state populations, such as A and B or Green and Red, was obtained from the relative signal intensities of nuclear spin pairs experiencing distinct NMR frequencies (chemical shifts) in the A- and B- (or Green and Red) states. As NMR intensities are also influenced by spin relaxation depending on the local dynamics of the nuclear spin pairs that might be different in the various conformational states, it is important to derive this population information for a number of different sites ensuring that they are not significantly perturbed by differential dynamics. In practice, relative peak intensities were measured for 10 to 20 well resolved ^{1}H-^{15}N and/or ^{1}H-^{13}C correlation peaks. Intensity ratios that differ by more than two standard deviations from the mean were discarded from further analysis, and the reported state populations correspond to the average value computed for the remaining sites.

Sample preparation for ensemble fluorescence microscopy

5 µL of purified FPs ~0.25 M concentration were added to 43 µL of buffer (1.5 mM Tris-HCl at pH 7) and 50 µL of 30% Acrylamide/Bis-acrylamide (29:1) solution. Acrylamide polymerization was started by adding 1 µL of 10% Ammonium persulfate (Sigma) and 1 µL of TEMED (Euromedex). The sample was thoroughly mixed and 10 µL of the mixture was spread between two glass coverslips pre-cleaned in a UV ozone cleaning system (HELIOS 500, UVOTECH Systems).

Ensemble fluorescence microscopy experiments

The data were acquired on a home-built setup based on an Olympus IX81 inverted microscope equipped with a 100x 1.49 NA oil-immersion apochromatic objective lens (Olympus, Japan). Widefield illumination was achieved by focusing the diode-pumped solid state 405-nm (CrystaLaser, USA) and 488-nm (Oxxius, France) laser beams to the back focal plane of the objective. Fluorescence images were acquired with an Evolve 512 back-illuminated EMCCD camera (Photometrics, USA) controlled by the Micro-Manager software.

Ensemble-level switching data were recorded in 1s frames consisting of 20 ms of fluorescence readout by weak 488-nm light (0.15 W/cm^2), 20 ms of dark-time, 400 ms of exposure to actinic light at either 488, or 405 nm (0.26 W/cm^2) followed by a 560 ms of dark-time. FPs were first off-switched for 600 seconds (i.e. 600 frames) under 488-nm light, and subsequently switched-on by 405-nm light for 50 seconds. The off-switching by 488 nm and subsequent back-switching by 405 nm was repeated for one more cycle. For certain experiments after the second 405 nm application a dark-time of 90 mins was inserted, followed by another off-switching period (see results). Analysis of switching cycles was performed on small, homogenous regions of the samples. All experiments were triplicated.

pKa measurement of the switched-off state

A stock solution of Green mEos4b was switched off by direct illumination for 30 minutes with a 488-nm diode-pumped solid-state laser (CrystaLaser, USA) and mixed regularly. This solution was then divided into five aliquots, and individually buffered to pH 6.0 (MES), 7.0 (HEPES), 8.0 (HEPES), 9.0 (CHES) and 10.0 (CAPS), using a final buffer concentration of 100 mM.

Absorption and emission spectra of each of the samples were measured immediately after preparation and buffer adjustment. The signals of the anionic absorption band and of the fluorescence emission do not show any significant evolution over the probed pH range, demonstrating that the pKa of the *E(trans)* form of mEos4b is higher than 10.

Photophysical simulations

Simulations were performed with SMIS[9] using the mEos4b photophysical model depicted in Figure 7. To simplify the simulations, we assumed that photoconversion only occurs from the neutral (protonated) Green A-state. The absorption spectrum of the mEos4b Green state was extracted from FPbase (https://www.fpbase.org/),[10] while absorption spectra of the

neutral states were taken from those of mEos2.[11] The thermal rates of A-B state exchange in the dark were obtained from the NMR data (k_{ex}=k_{AB}+k_{BA} = $2e^{-2}$ s^{-1}, and P_A/P_b= k_{BA}/k_{AB} = 1.33). The photoconversion quantum yield from the green neutral state and the photobleaching quantum yield from the green anionic state were based on those reported for other mEos variants. [12–14] For the simulation, the green A and B states were assumed to have the same pKa (5.5) and Hill coefficient (0.74),[15] and the same brightness. First, the quantum yields of the A-B state exchange were adjusted to reproduce UV-light-dependence of the A/B-state population ratio observed by NMR. Second, the bleaching quantum yields from the Green neutral (A and B) states were adjusted in order to reproduce the UV-light-dependence of the photoconversion efficiency observed by NMR. Simulations were performed for 10^4 minutes under continuous illumination with 2.5 to 25 mW/cm^2 405-nm light.

SUPPLEMENTARY TABLE S1

	GREEN A ANIONIC	GREEN A NEUTRAL	GREEN B ANIONIC	GREEN B NEUTRAL	BLEACHED GREEN	RED NEUTRAL
**GREEN A ANIONIC**	-	-	0.0086*	-	2.5e^{-6}	-
**GREEN A NEUTRAL**	-	-	-	0.0086* **0.3015**	**3.5e^{-6}**	5.0e^{-4}
**GREEN B ANIONIC**	0.0114*	-	-	-	2.5e^{-6}	-
**GREEN B NEUTRAL**	-	0.0114* **0.0707**	-	-	**5.0e^{-4}**	-

Quantum yields and thermal inter-conversion rates (*) used in the SMIS simulations of mEos4b photoconversion. These values allow reproducing the NMR results, showing that the proposed photoconversion and photobleaching model is in qualitative agreement with our NMR observations. Fitted values are shown in bold. The obtained values of the photobleaching quantum yields from the A and B neutral states should be considered with care in view of the simplifications assumed.

SUPPLEMENTARY FIGURES

Figure S1: *PCFPs derived from Lobophyllia hemprichii EosFP*

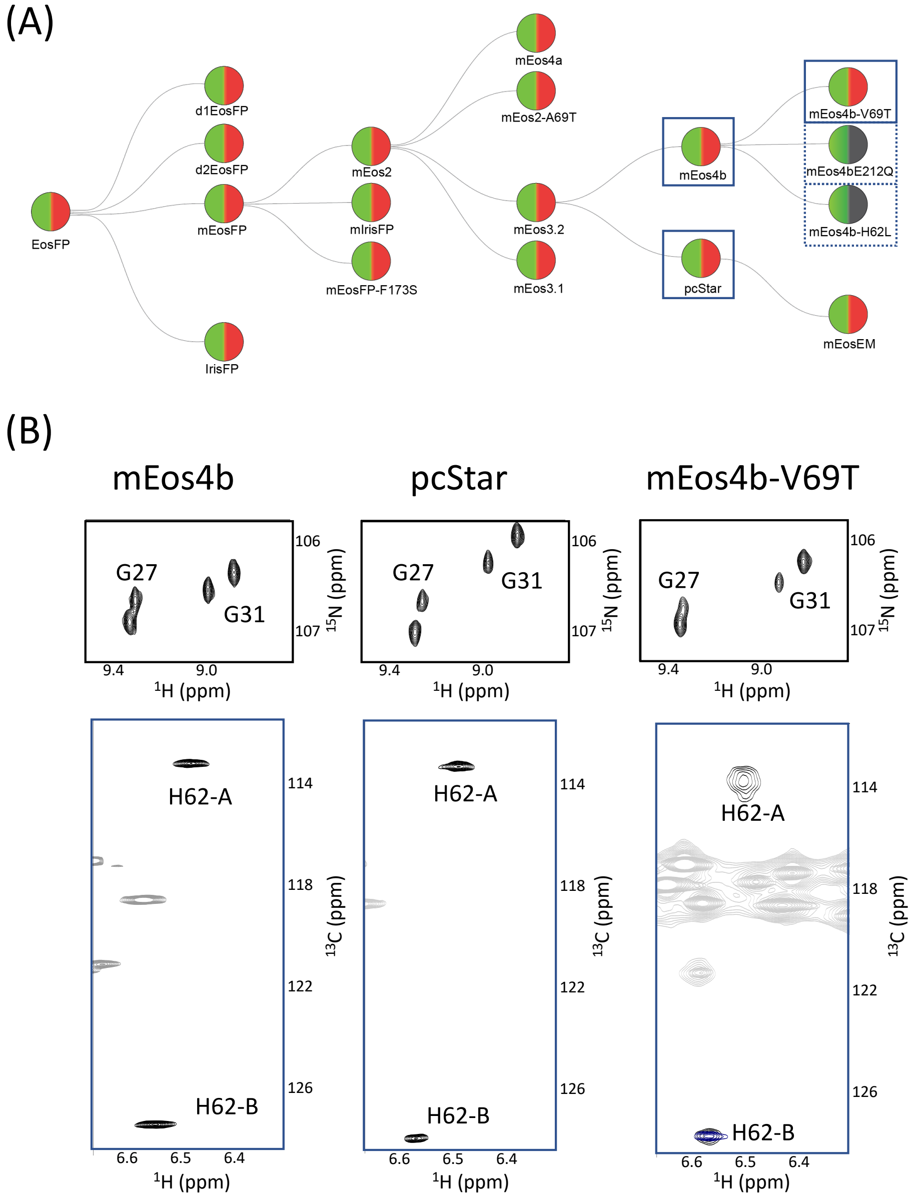

(A) Evolutionary tree of photoconvertible fluorescent proteins (PCFPs) derived from *Lobophyllia hemprichii* EosFP. The variants used in this study are indicated by boxes, including the 2 mutants (E212Q and H62L) that are not capable to photoconvert. (B) NMR spectra of mEos4b-WT, pcStar, and mEos4b-V69T highlighting the similar conformational heterogeneity present in all 3 PCFP variants. The upper panel shows ^{1}H-^{15}N correlations detected for the 2 amide sites G27 and G31, while the lower spectra correspond to the CD_2-H site of H62 in the A- and B-states. Note that for mEos4b-V69T strong NMR line broadening is detected for H62 in the A-state, and the corresponding cross peak is only visible in a non-constant-time (CT) HSQC experiment.

Figure S2: *Green A-B-state chemical shift differences*

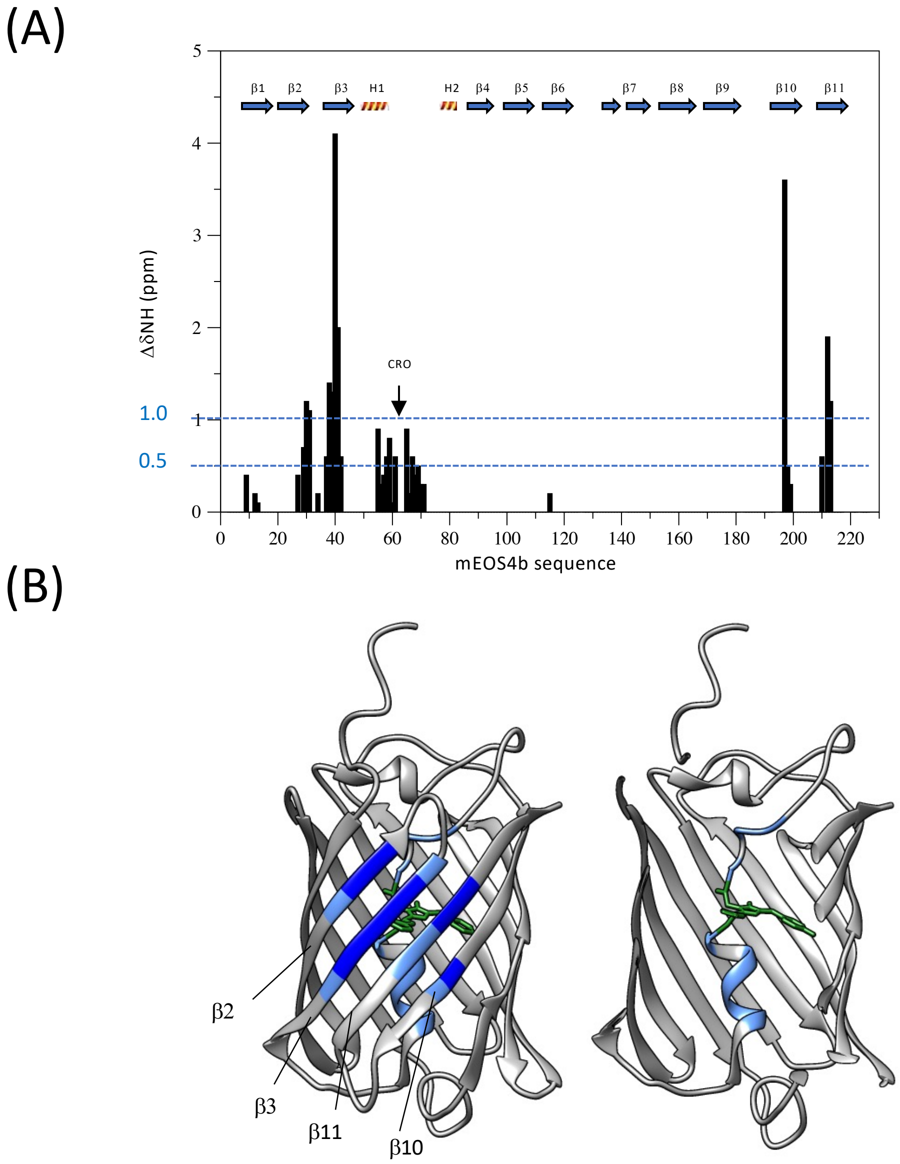

[1]H-^{15}N chemical shift differences between the Green A- and B-states of mEos4b. $\Delta\delta_{HN}$ values have been computed as $\sqrt{(\Delta N^2 + 10\Delta H^2}$ and are plotted in (A) as a function of the mEos4b peptide sequence. (B) The largest chemical shift changes between the A- and B-states are color-coded on the crystal structure of mEos4b Green-state (dark blue: $\Delta\delta_{HN} > 1.0$ ppm; light blue: $0.5 \text{ ppm} < \Delta\delta_{HN} < 1.0$ ppm).

Figure S3*: NMR signature of p-HBI chromophore*

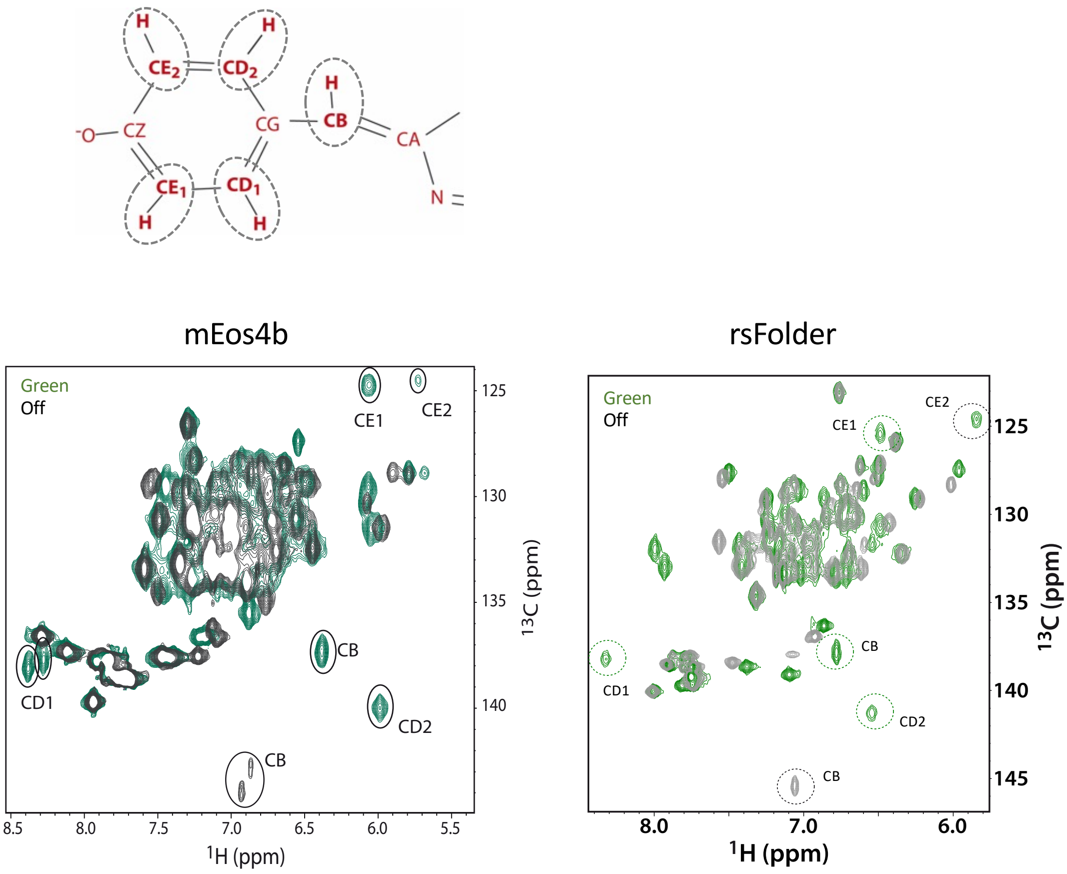

Aromatic ^{1}H-^{13}C Best-HSQC spectra of the PCFP mEos4b (left) and the RSFP rsFolder (right). Spectra recorded in the dark (Green state) and under continuous 448-nm illumination (Off-state) are color-coded and overlaid. The correlation peaks corresponding to ^{1}H-^{13}C spin pairs of the chromophore's phenol moiety and the methine bridge (upper graph) are highlighted by circles and annotated. The NMR spectral signature of the p-HBI chromophore is very similar despite the low sequence identity of the 2 proteins. The identification and assignment of chromophore ^{13}C-^{1}H correlations was facilitated by similar spectral signatures of the p-HBI chromophore in mEos4b and rsFolder, despite the low sequence identity between these FPs. rsFolder is a hydrozoan-derived RSFP,[16] for which we have recently performed extensive NMR investigations.[8,17]

Figure S4: *Chromophore NMR resonance assignment and ring flips*

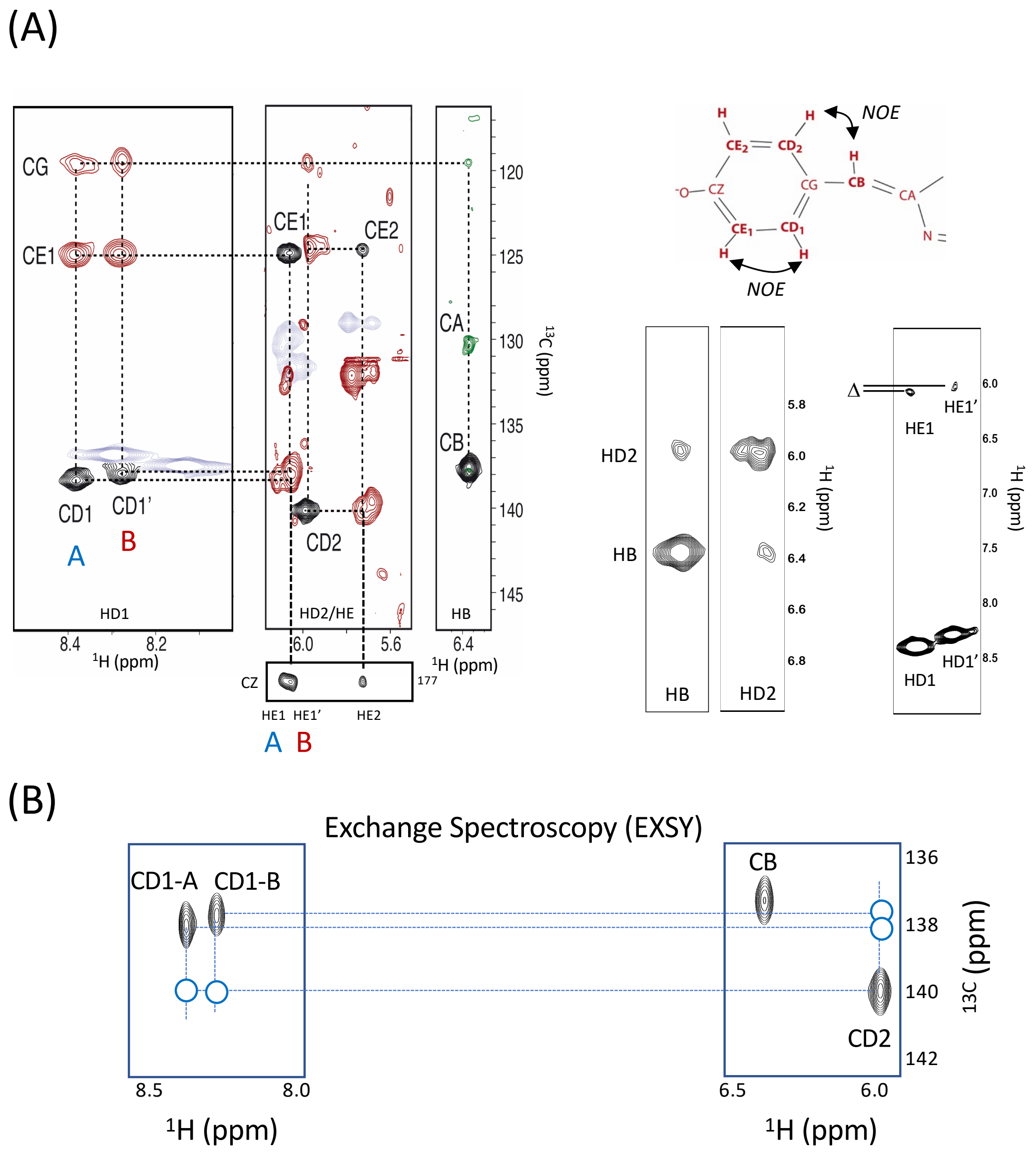

NMR spectra acquired for chromophore resonance assignment purposes. (A) Different ^{1}H-^{13}C correlation spectra are superposed (left panel) and color-coded to highlight the connectivity information obtained from these experiments: aromatic Best-HSQC (black), Best-HCC (red) and Best-HBCBCA (green). The chromophore resonances are annotated. Unambiguous stereospecific assignments of ^{13}CD and ^{13}CE sites are obtained from NOESY-type experiments (right panel). (B) 2D C_zH_z-EXSY spectra recorded to quantify chromophore ring-flip rates of mEos4b at pH 8.5 (anionic chromophore). The mixing delay was set to 100 ms. No exchange correlation peaks could be detected in these spectra (detection limit ~1% of diagonal peak) at the expected spectral positions (highlighted by blue circles), indicating that the chromophore ring flip rate constant is less than 1 s^{-1} in both, the A- and B-states.

Figure S5*: NMR chemical shift differences between WT A- B-states and E212Q mutant*

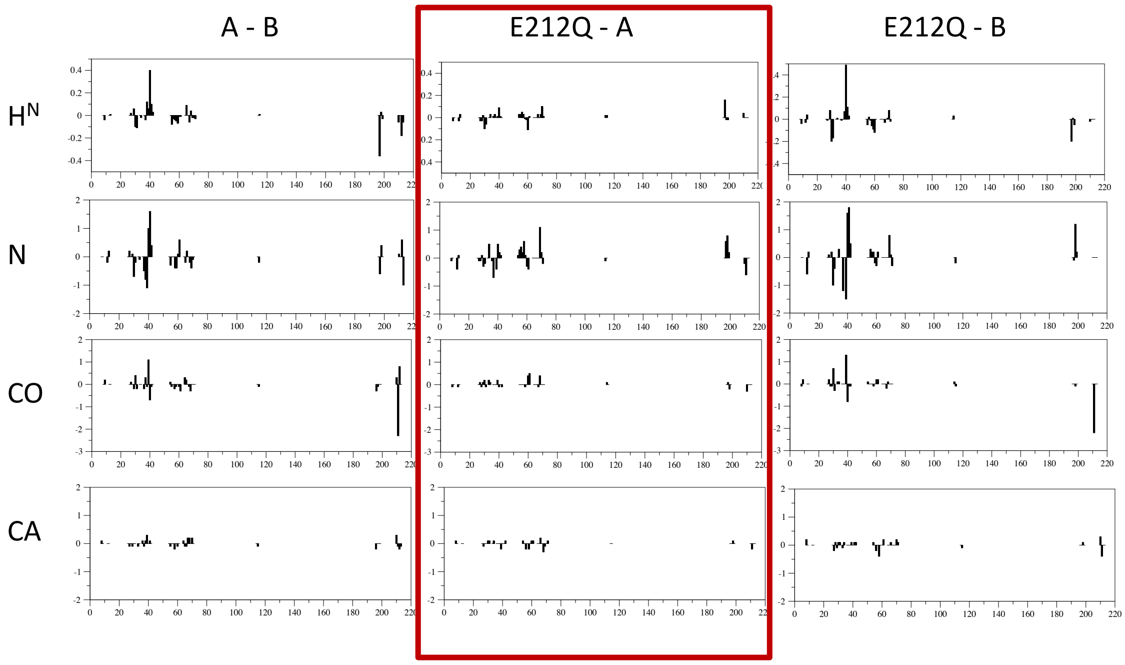

Differences in NMR chemical shifts between mEos4b-WT Green A- and B-states (left panel), mEos4b-E212Q and WT A-state (middle panel), and mEos4b-E212Q and WT B-state (right panel). The computed chemical shift differences for 4 backbone nuclei are plotted as a function of the peptide sequence. The CA chemical shifts are very similar in all cases, indicating little differences in secondary structure. Significant CO chemical shift differences are detected in certain peptide regions (especially close to E212) between the A- and B-states, indicative of differences in hydrogen bonding. The E212Q mutant almost perfectly matches the CO chemical shifts of the WT A-state.

Figure S6 *: UV-Vis absorbance spectra*

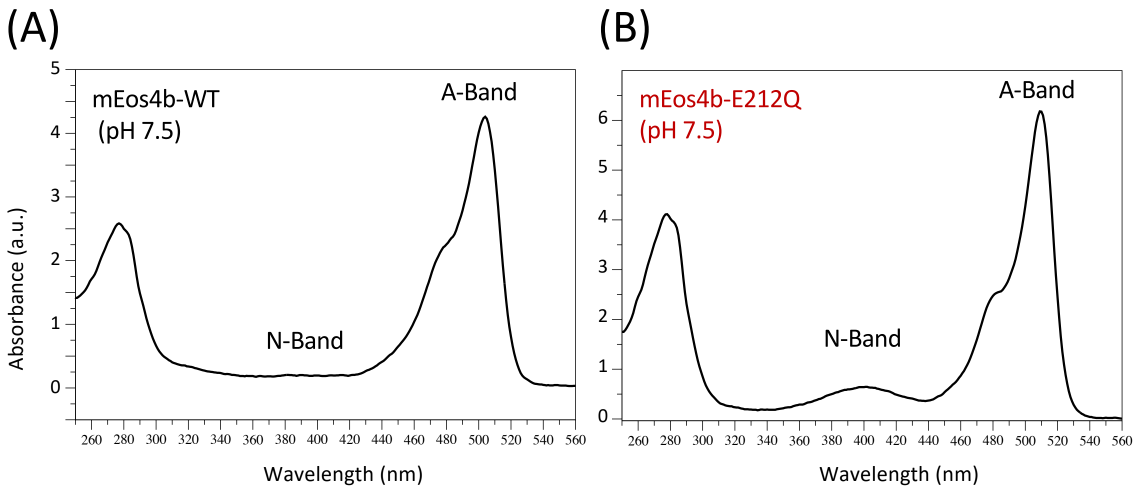

UV-Vis absorbance spectra of (A) mEos4b-WT (pH 7.5) and (B) mEos4b-E212Q (pH 7.5) measured at room temperature. The N- and A-bands, corresponding to the absorbance of a protonated and anionic chromophore species are annotated.

Figure S7*: Histidine H-bonding and dynamics in mEos4b-WT*

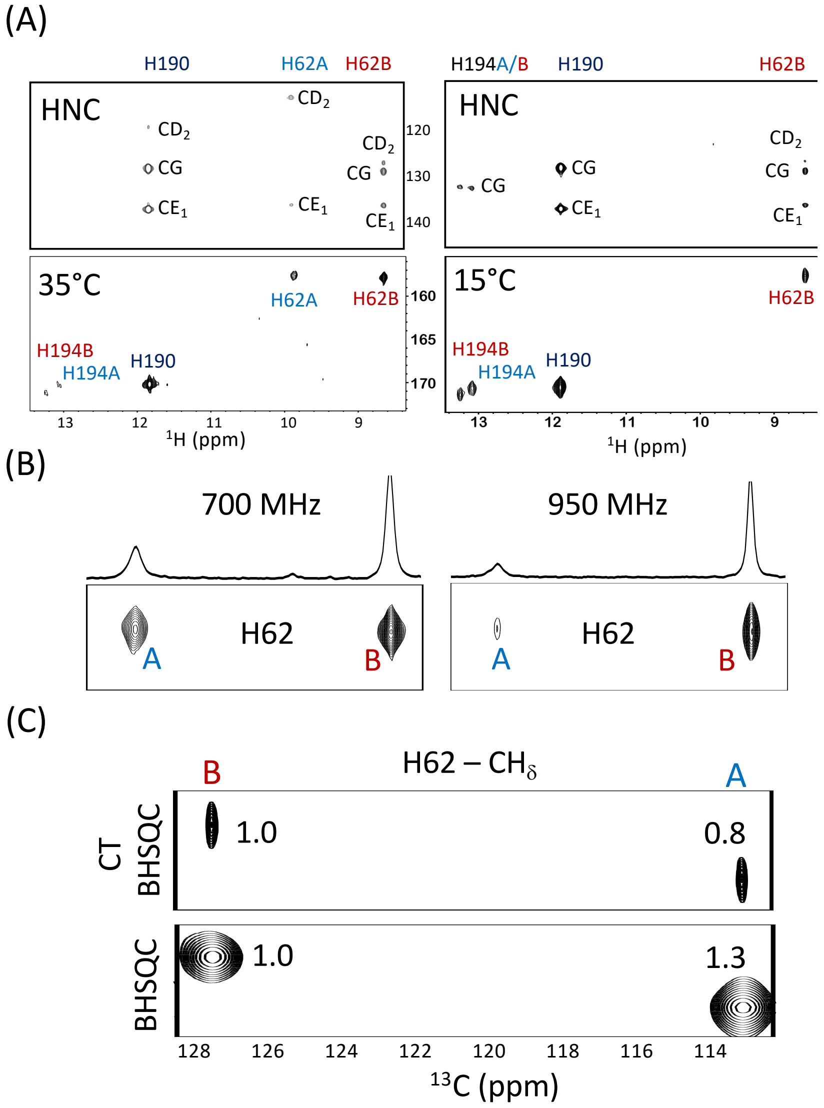

NMR spectra of mEos4b-WT (pH 7.5). (A) ^{1}H-^{15}N histidine correlation spectra of mEos4b recorded at 35°C and 15°C. The detected correlation peaks are annotated. Unambiguous resonance assignment was obtained from 2D Best-TROSY histidine HNC correlation spectra (plotted on top) that correlate the imidazole ^{1}H^{N} with adjacent ring ^{13}C. (B) Magnetic field dependence of H62 ^{1}H-^{15}N peak intensities in the A- and B-states. At a ^{1}H frequency of 950 MHz, the A-state peak is attenuated by a factor of ~2 with respect to the 700 MHz data (C) ^{1}H-^{13}C correlation peaks corresponding to the CD_2 site in H62 in the A- and B-states extracted from either a CT-BHSQC (top) or non-CT BHSQC (bottom) spectrum. The numbers indicate relative peak intensities of the A-state with respect to the B-state correlation. The observed intensity loss of the A-state (with respect to the B-state) peak during the additional relaxation delay in the constant-time experiment is explained by conformational exchange dynamics in the A-state.

Figure S8: *Hydrogen bonding in Green A- and B-states*

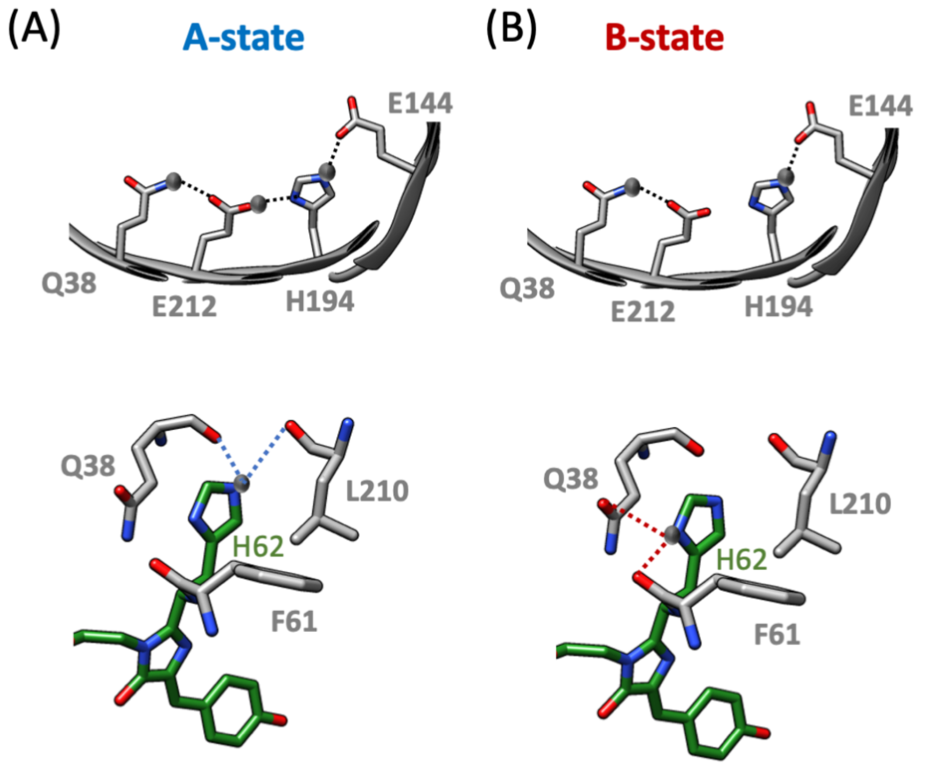

Differences in side chain protonation and hydrogen bonding patterns in the chromophore pocket of (A) Green A-state and (B) Green B-state. The NMR-identified hydrogens are indicated by grey balls, and H-bonds are shown as dashed lines. The potential H-bond acceptors are derived from the crystal structure (PDB 6GOY) as the closest oxygen or nitrogen atoms.

Figure S9*: Off A-B-state chemical shift differences*

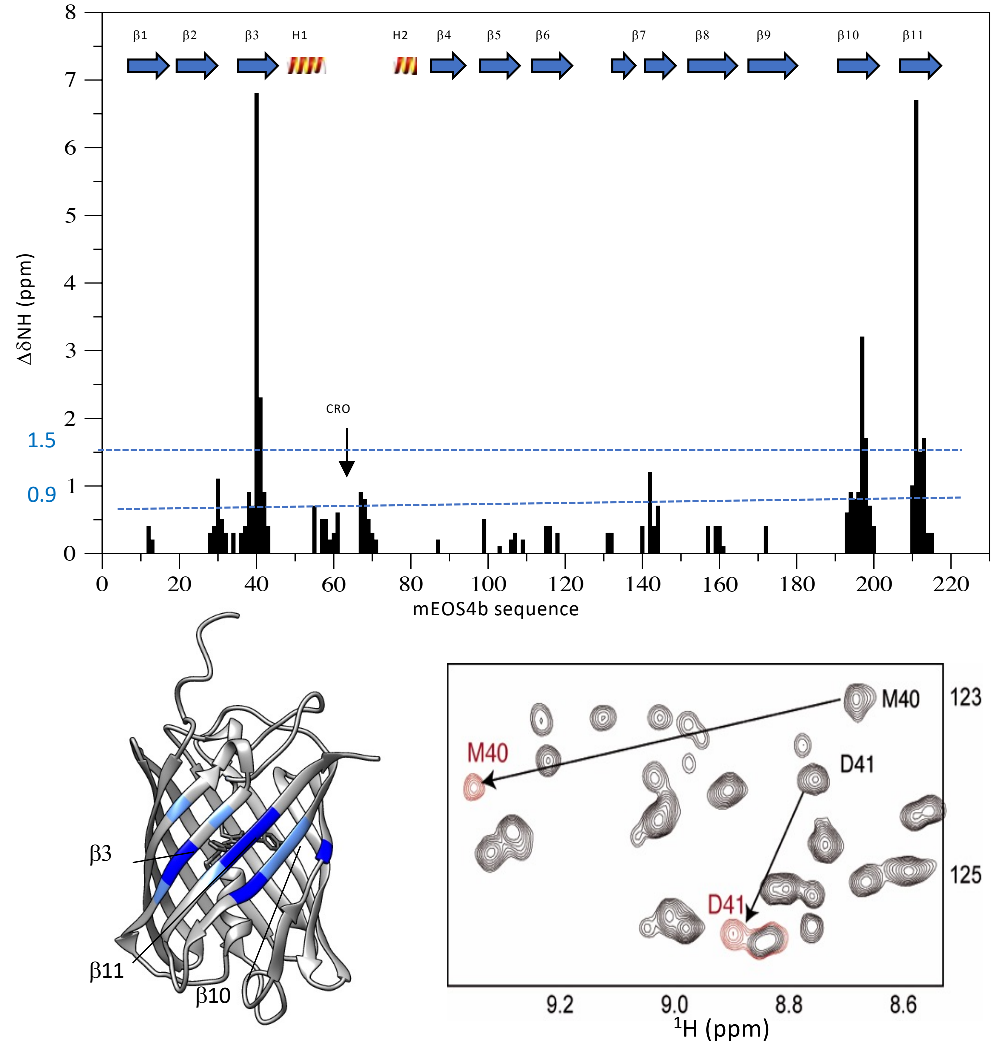

^{1}H-^{15}N chemical shift differences between the photoswitched Off-A and Off-B states of mEos4b. $\Delta\delta_{HN}$ values have been computed as $\sqrt{(\Delta N^2 + 10\Delta H^2}$ and are plotted as a function of the mEos4b peptide sequence (upper graph). The largest chemical shift changes between the A- and B-states are color-coded on the crystal structure of mEos4b Off-state (dark blue: $\Delta\delta_{HN} > 1.5$ ppm; light blue: 0.9 ppm $< \Delta\delta_{HN} <$ 1.5 ppm). In addition, part of a ^{1}H-^{15}N spectrum is shown highlighting the large chemical shift changes observed for M40 and D41 between the Off-A and Off-B states.

Figure S10: *Green mEos4B NMR spectra with/without UV illumination*

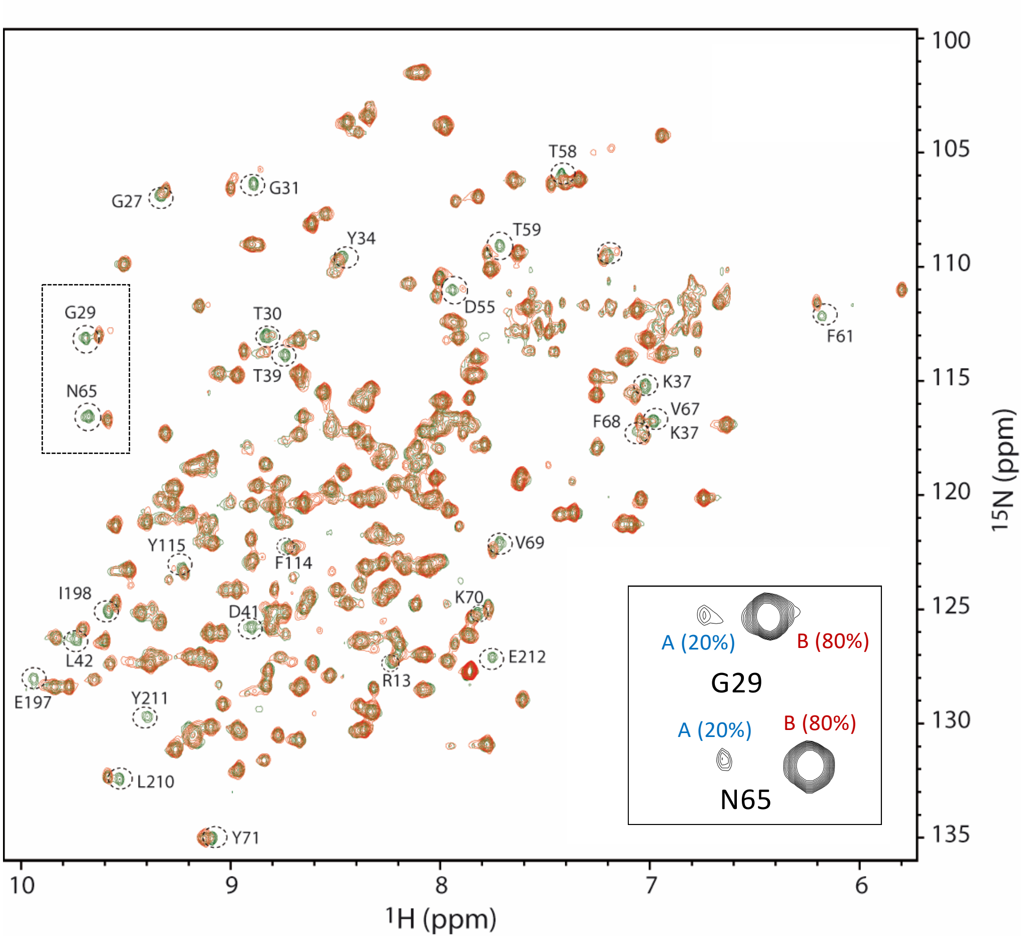

Superposition of ^{1}H-^{15}N correlation spectra of mEos4b recorded in the dark (green contours) and after short high-power UV (405-nm) illumination (red contours). Peaks that are only visible in the Green spectrum (highlighted by dashed circles) correspond to the A-state. The insert shows a small part of the spectrum recorded under high-power UV plotted at lower contour levels, in order to observe the residual peak intensity of ~20% detected for the A state.

Figure S11*: Fluorescence data of mEos4b photoswitching*

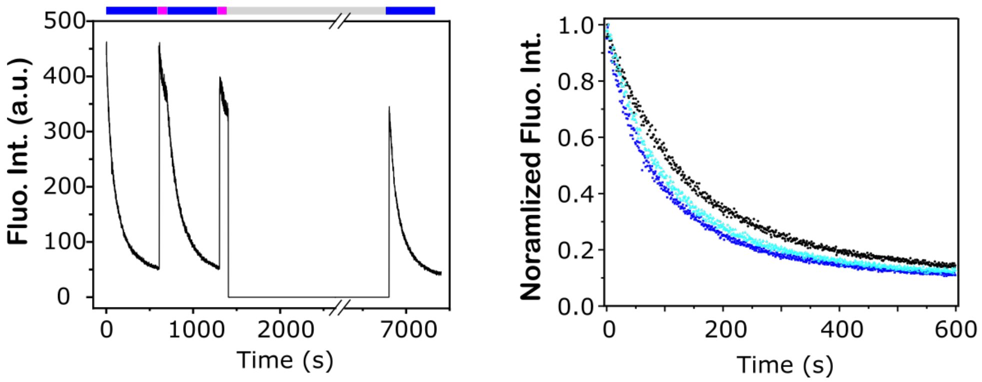

Additional ensemble fluorescence data illustrating the reversibility of altered photo-switching kinetics after UV illumination by inserting a thermal relaxation delay. Raw traces of fluorescent switching cycles are shown in the left panel, with the illumination scheme used on top. In total, three cycles of 488-nm induced off-switching of mEos4b were performed; the second and third cycles were separated by a 90 -min dark period, during which, the lasers and camera were turned-off. Normalized off-switching traces are superposed in the right panel: first cycle (blue), second cycle (black), and third cycle (cyan).

Figure S12*: pKa measurement of photoswitched Off state*

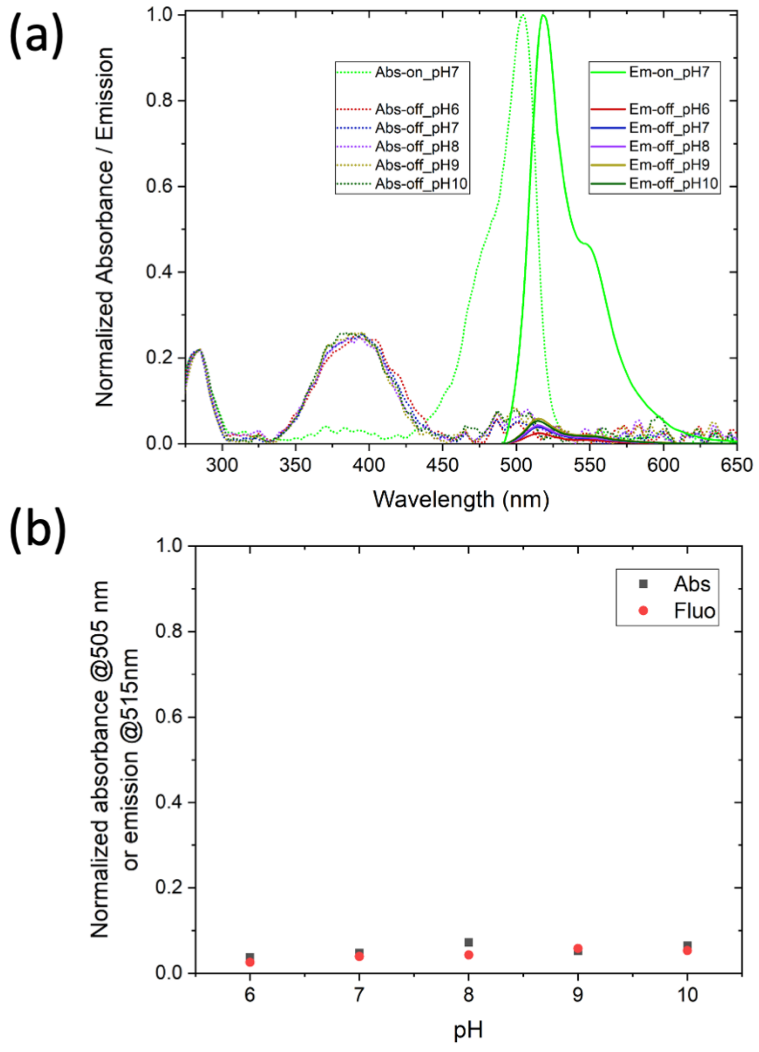

(a) Absorbance (dotted lines) and emission (plain lines) spectra of mEos4b in its initial Green state at pH 7.0 (green) and after off-switching and buffering at pH values ranging from 6 to 10. (b) Evolution of the peak absorbance of the anionic band (black squares) and peak emission (red dots) of the Off-state from pH 6 to 10.

Figure S13: *NMR spectrum of Red mEos4B*

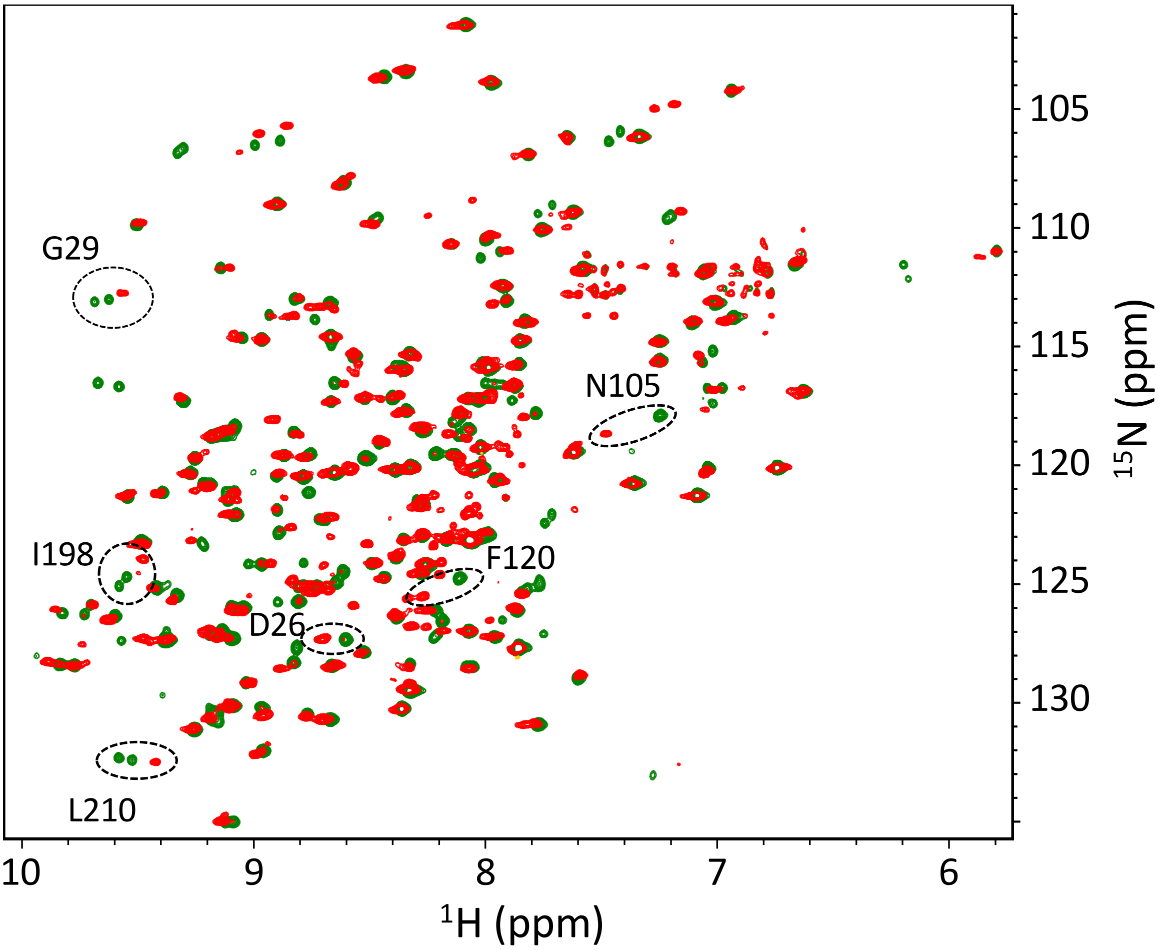

Superposition of ^{1}H-^{15}N correlation spectra of mEos4b Green (green contours) and Red (red contours) states. Correlation peaks (residues) that were used for the analysis of photoconversion kinetics are annotated.

Figure S14: *NMR photoconversion kinetics*

In-situ NMR photoconversion kinetics under continuous UV (405-nm) illumination using different light power densities (measured at the top of the NMR sample): (a) 2.5 mW/cm², (b) 3 mW/cm², (c) 5 mW/cm², (d) 13 mW/cm², and (e) 25 mW/cm². The different graphs show NMR peak intensities (arbitrary unites) as a function of illumination time (in min). The straight lines correspond to a global fit of all Green-state decay and Red-state buildup data for a given power density to the following model. Green-state decay: $G(t) = G_0*(0.35 \exp(-t/T_1) + 0.65 \exp(-t/T_2))$ and Red-state build-up $R(t) = R_0*(0.35 (1-\exp(-t/T_1)) + 0.65 (1-\exp(-t/T_2)))* \exp(-t/T_{BL})$, with T_1, T_2 and T_{BL} global fit parameters. The relative amplitudes of the kinetic phases were optimized in an initial step by a grid-search procedure, and then fixed to allow a quantitative comparison of the light-power dependence of the kinetic rates (time constants).

(a) The fitted time constants are: $T_1 = 122 \pm 10$ min; $T_2 = 776 \pm 30$ min; $T_{BL} = 10000 \pm 1000$ min

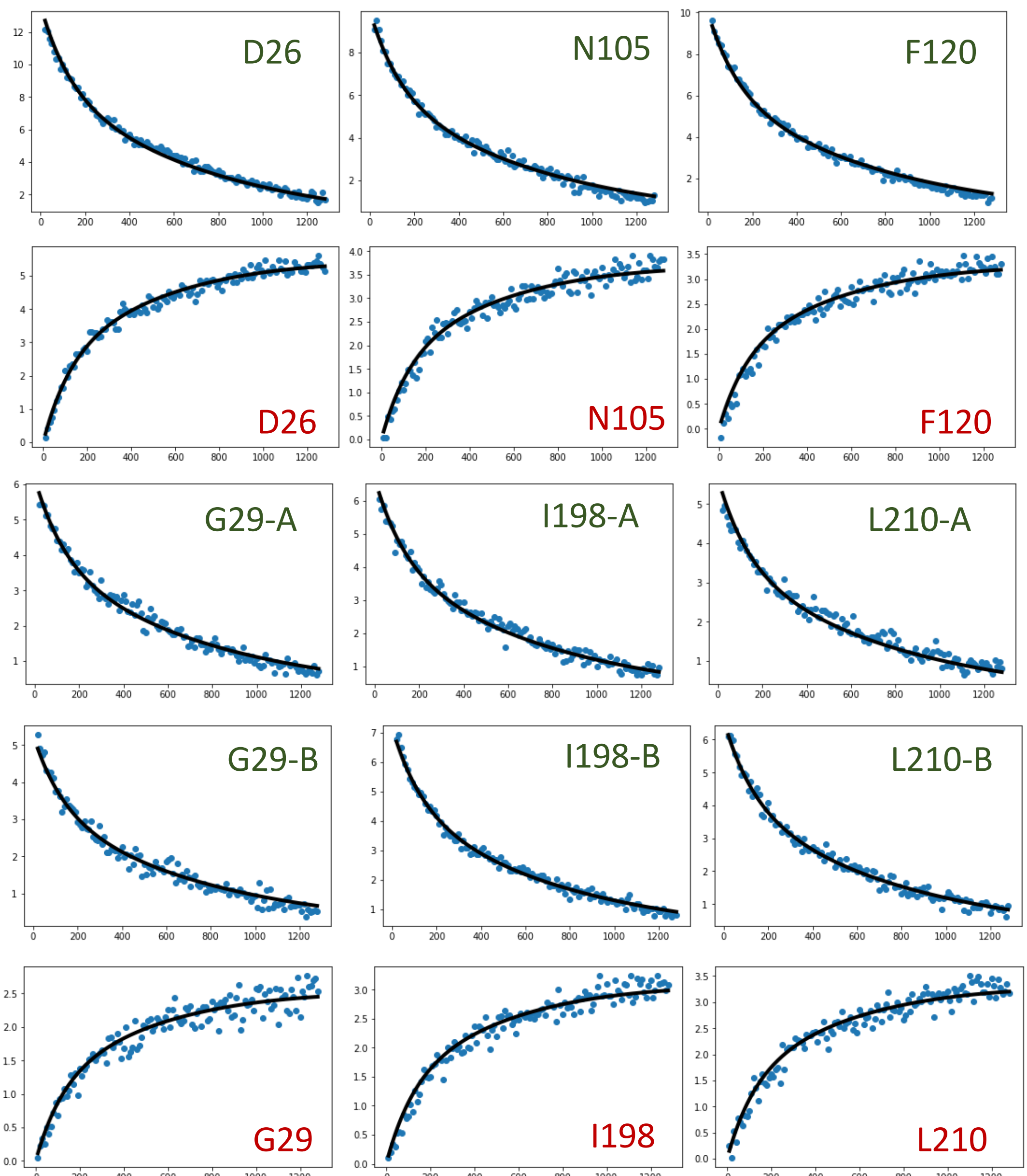

(b) The fitted time constants are: $T_1 = 100 \pm 10$ min; $T_2 = 737 \pm 30$ min; $T_{BL} = 8000 \pm 800$ min

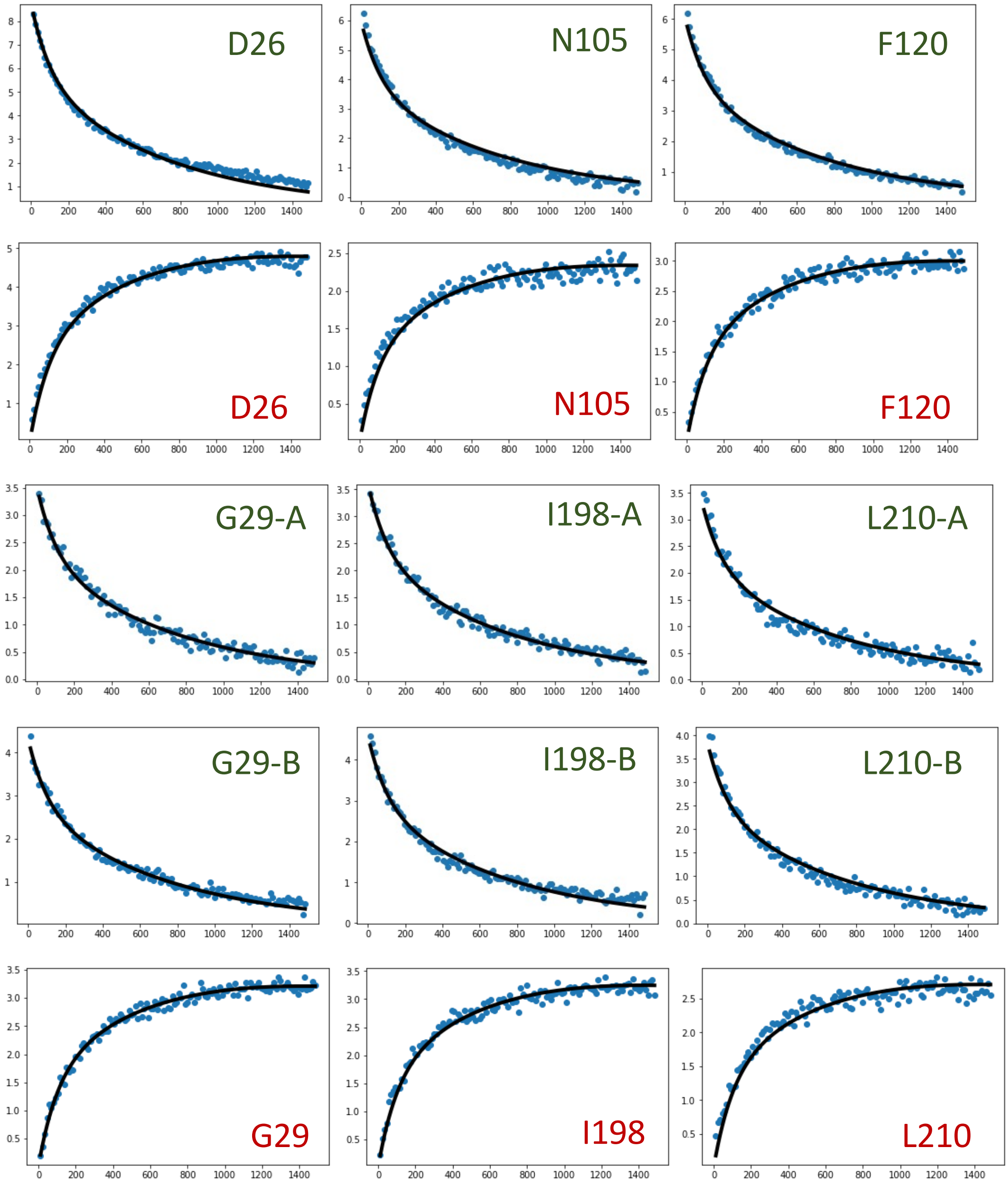

(c) The fitted time constants are: $T_1 = 61 \pm 6$ min; $T_2 = 410 \pm 20$ min; $T_{BL} = 4400 \pm 400$ min

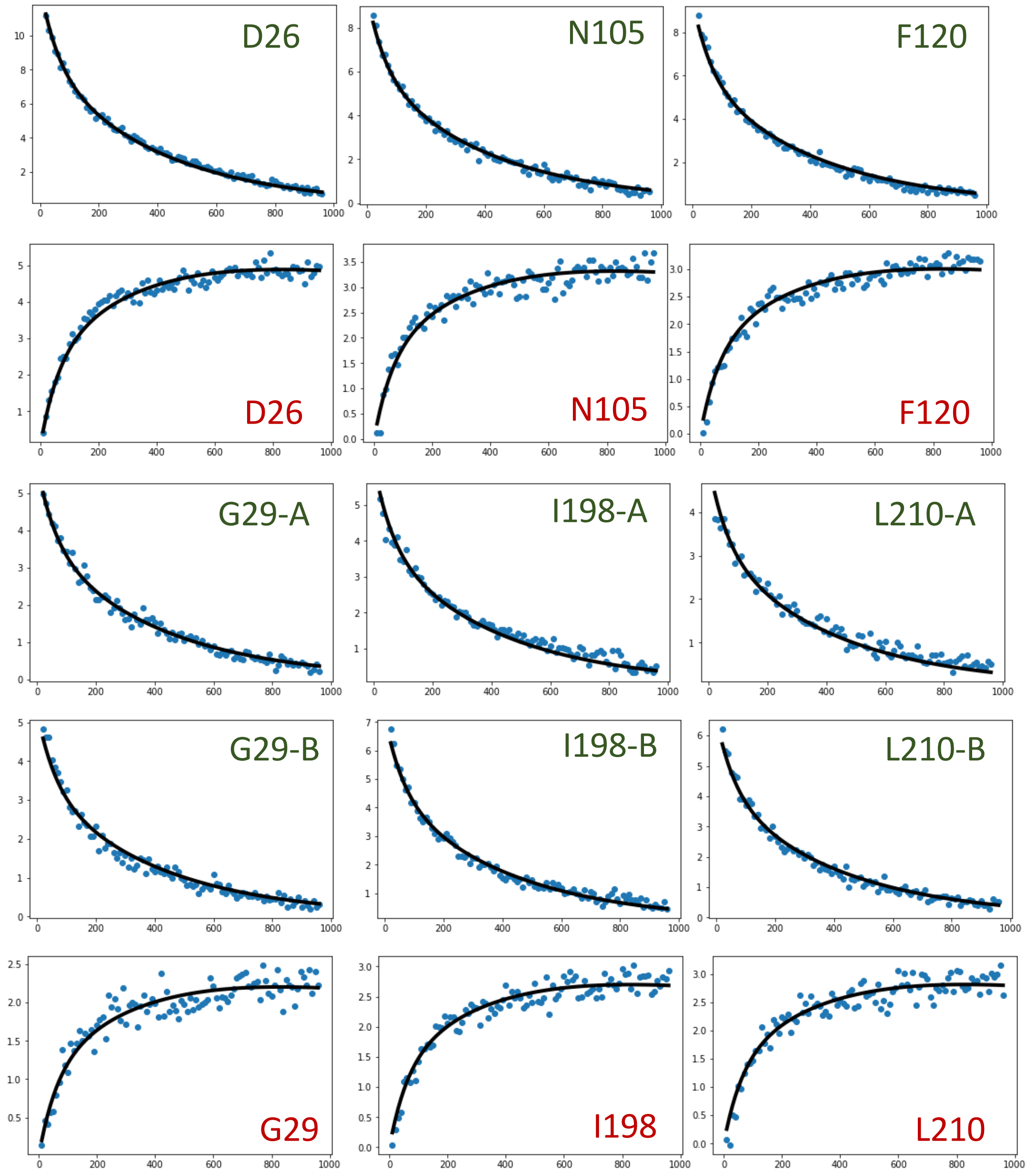

(d) The fitted time constants are: $T_1 = 25 \pm 3$ min; $T_2 = 190 \pm 10$ min; $T_{BL} = 1600 \pm 160$ min

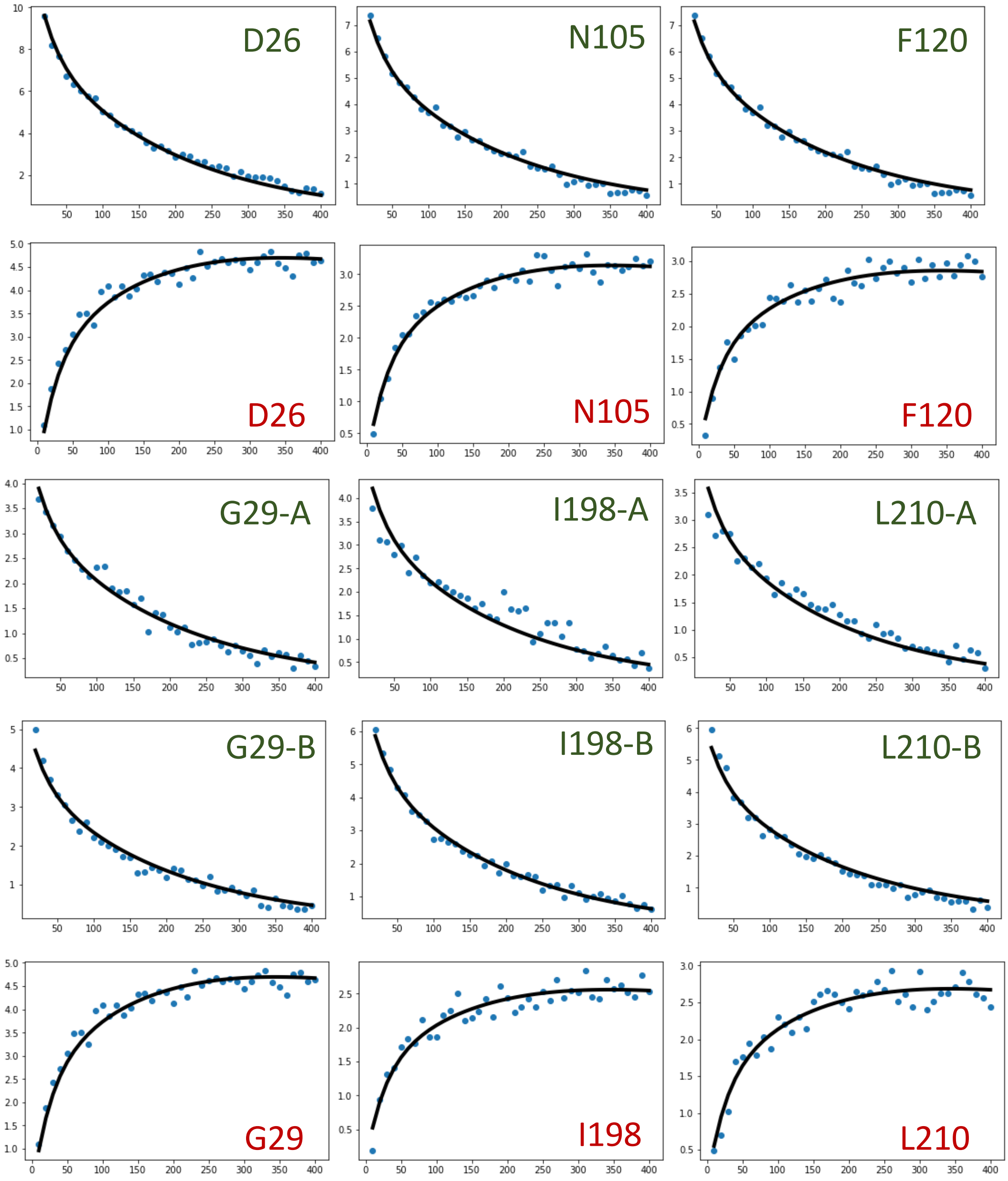

(e) The fitted time constants are: $T_1 = 12 \pm 3$ min; $T_2 = 75 \pm 10$ min; $T_{BL} = 900 \pm 100$ min

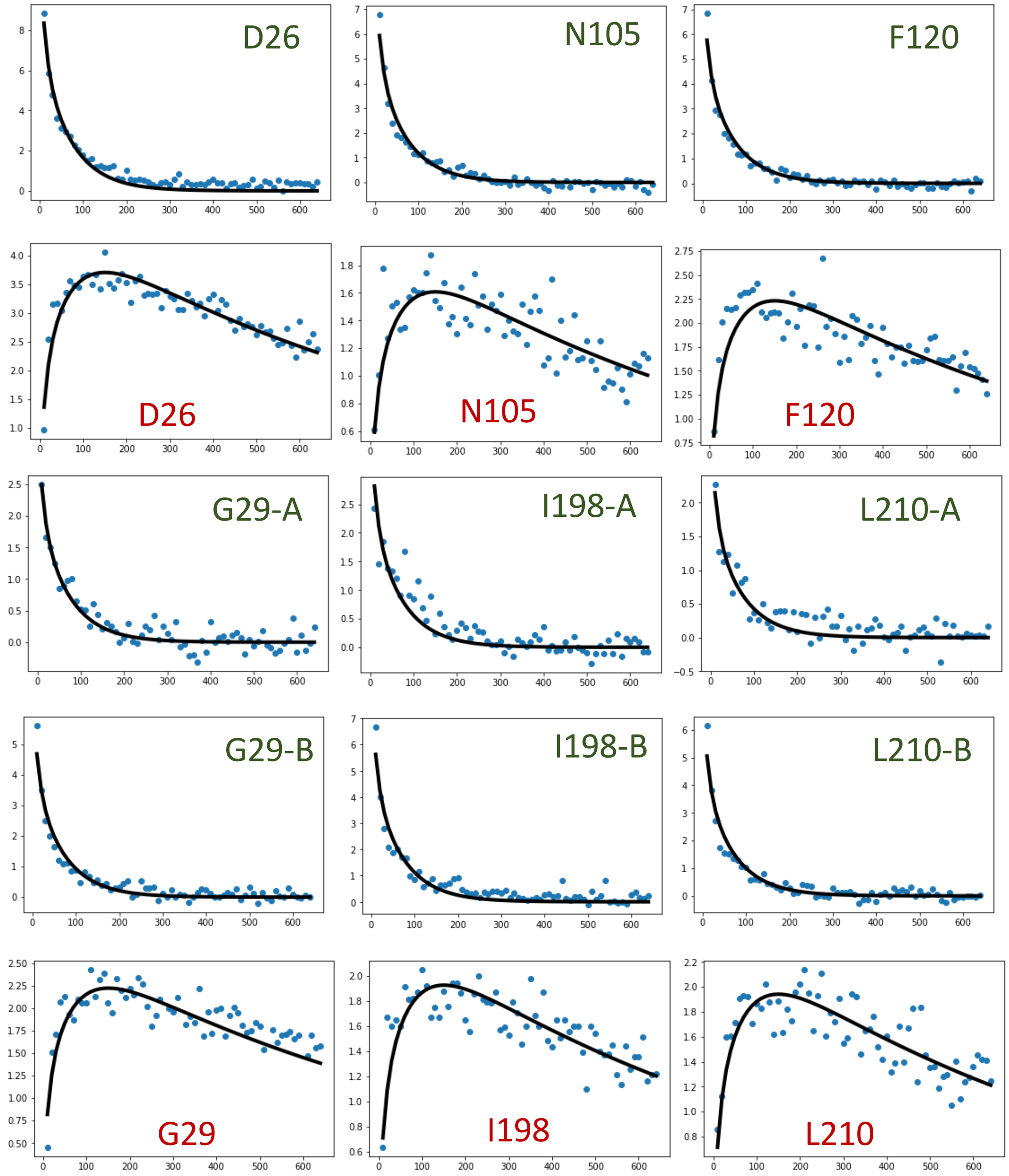

Figure S15: *SMIS simulations of Green-to-Red photoconversion*

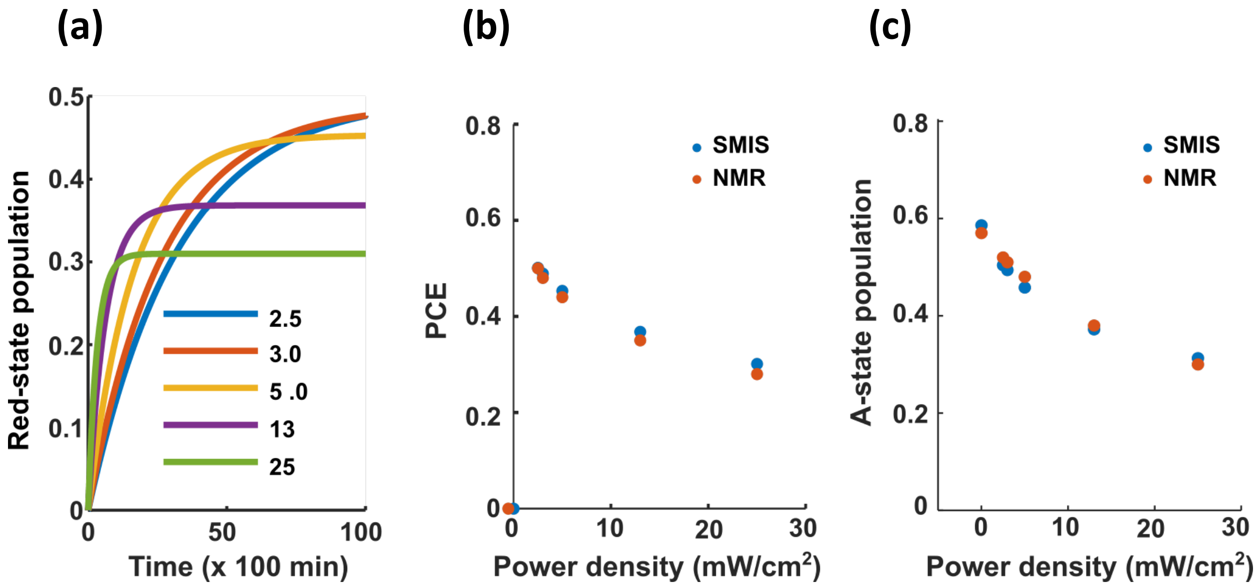

Photophysical simulations using SMIS [9]: (a) Simulated Red-state build-up kinetics based on the model depicted in Fig. 8B and kinetic rates (quantum yields) as given in Table S1. Note that no Red-state bleaching was included in these simulations. Build-up curves are computed for power densities in the range 2.5 to 25 mW/cm2. Comparison of (b) photoconversion efficiencies (PCE) and (c) A-state populations as a function of UV power density extracted from the NMR data and SMIS simulations. For the simulations, the inter-conversion rates between the A and B states were adjusted to reproduce the NMR-observed thermal and light-dependent A/B state population ratio and exchange kinetics. The photoconversion rates from the A state and photobleaching from the B state were set to match the experimentally observed Red-state build-up kinetics and photoconversion efficiency as a function of UV power density. We did not attempt to reproduce in our simulations the time scale of photoconversion, nor the bi-phasic kinetics observed in the NMR data, notably because those are likely influenced by the inhomogeneous NMR sample illumination. Furthermore, we assumed in our simulations that the chromophore in the A- and B-states shows the same pKa and absorption spectrum. Even small differences in these properties between the A- and B-states may lead to significant changes in the computed photoconversion and photobleaching kinetics.

Figure S16*: Proposed main photoconversion model*

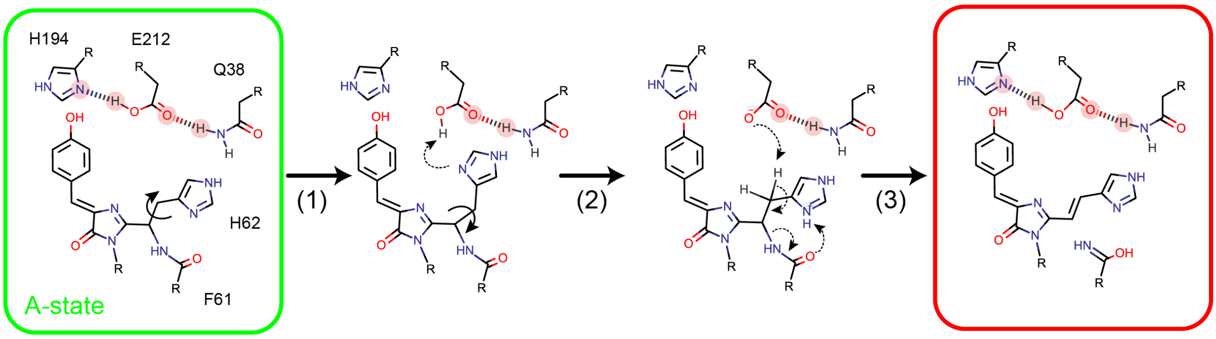

Proposed main photoconversion pathway of mEos4b starting from the Green A-state, adapted from Kim et al,[18] in agreement with our NMR observations. Step 1: transient ground-state breakage of the hydrogen bond between H194 and dE212, and rotamer adjustment of H62 that positions the deprotonated ND_1 in proximity of the protonated E212 side chain. Step 2: UV photon absorption leads to excited state proton transfer (ESPT) from the E212 carboxyl group to H62-ND_1, followed by rotamer adjustment of H62. Step 3: Photoconversion then proceeds via a concerted β-elimination mechanism involving backward proton shuttling from H62-CB to E212. The necessity for a labile proton at residue E212 is in line with the fact that photoconversion is abolished in the E212Q mEos4b mutant.

REFERENCES

[1] W. F. Vranken, W. Boucher, T. J. Stevens, R. H. Fogh, A. Pajon, M. Llinas, E. L. Ulrich, J. L. Markley, J. Ionides, E. D. Laue, *Proteins Struct. Funct. Genet.* **2005**, *59*, 687–696.

[2] A. Favier, B. Brutscher, *J. Biomol. NMR* **2011**, *49*, 9–15.

[3] P. Schanda, B. Brutscher, *J. Am. Chem. Soc.* **2005**, *127*, 8014–8015.

[4] P. Schanda, E. Kupce, B. Brutscher, *J. Biomol. NMR* **2005**, *33*, 199–211.

[5] N. E. Christou, B. Brutscher, *J. Biomol. NMR* **2018**, *72*, 115–124.

[6] Z. Solyom, M. Schwarten, L. Geist, R. Konrat, D. Willbold, B. Brutscher, *J. Biomol. NMR* **2013**, *55*, 311–21.

[7] A. Favier, B. Brutscher, *J. Biomol. NMR* **2019**, *73*, 199–211.

[8] N. E. Christou, I. Ayala, K. Giandoreggio-Barranco, M. Byrdin, V. Adam, D. Bourgeois, B. Brutscher, *Biophys. J.* **2019**, *117*, 2087–2100.

[9] D. Bourgeois, *Commun. Biol.* **2023**, *6*, 1–13.

[10] T. J. Lambert, *Nat. Methods* **2019**, *16*, 277–278.

[11] R. Berardozzi, V. Adam, A. Martins, D. Bourgeois, *J. Am. Chem. Soc.* **2016**, *138*, 558–565.

[12] R. Ando, H. Hama, M. Yamamoto-Hino, H. Mizuno, A. Miyawaki, *Proc. Natl. Acad. Sci. U. S. A.* **2002**, *99*, 12651–12656.

[13] S. Boehme, G. Desfonds, K. Nienhaus, M. J. Field, V. Adam, J. Wiedenmann, S. Mcsweeney, G. U. Nienhaus, D. Bourgeois, *Proc. Natl. Acad. Sci.* **2008**, *105*, 18343–18348.

[14] D. Thédié, R. Berardozzi, V. Adam, D. Bourgeois, *J. Phys. Chem. Lett.* **2017**, *8*, 4424–4430.

[15] M. G. Paez-Segala, M. G. Sun, G. Shtengel, S. Viswanathan, M. A. Baird, J. J. Macklin, R. Patel, J. R. Allen, E. S. Howe, G. Piszczek, et al., *Nat. Methods* **2015**, *12*, 215–218.

[16] M. El Khatib, A. Martins, D. Bourgeois, J. Colletier, V. Adam, M. El Khatib, A. Martins, D. Bourgeois, J. Colletier, V. Adam, *Sci. Rep.* **2016**, *6*, 18459.

[17] N. E. Christou, K. Giandoreggio-Barranco, I. Ayala, O. Glushonkov, V. Adam, D. Bourgeois, B. Brutscher, *J. Am. Chem. Soc.* **2021**, *143*, 7521–7530.

[18] H. Kim, T. Zou, C. Modi, K. Dörner, T. J. Grunkemeyer, L. Chen, R. Fromme, M. V. Matz, S. B. Ozkan, R. M. Wachter, *Structure* **2015**, *23*, 34–43.
